# Phylogenetic analysis of paired breast carcinomas identifies genetic events associated with clonal recurrence and invasive progression

**DOI:** 10.1002/path.6461

**Published:** 2025-10-30

**Authors:** Tanjina Kader, Maia Zethoven, Sakshi Mahale, Hugo Saunders, Lauren Tjoeka, Rebecca Lehmann, Madawa W Jayawardana, Jia‐Min Pang, Dorothea Lesche, Neeha Rajan, Timothy Semple, Jue Er Amanda Lee, Richard Lupat, David J Byrne, Siobhan Hughes, Hoa Nguyen, Siqi Lai, Maree Pechlivanis, Olivia Craig, Lisa Devereux, Eloise House, Sureshni I Jayasinghe, Tom L Kaufmann, Roland F Schwarz, Andrew R Green, Islam M Miligy, Margaret Cummings, Sunil Lakhani, Ian G Campbell, Emad Rakha, Stephen B Fox, G Bruce Mann, Kylie L Gorringe

**Affiliations:** ^1^ Peter MacCallum Cancer Centre Melbourne Australia; ^2^ The Sir Peter MacCallum Department of Oncology The University of Melbourne Parkville Australia; ^3^ The Breast Service The Royal Women's Hospital Melbourne Australia; ^4^ Department of Anatomical Pathology St Vincent's Hospital Melbourne Fitzroy VIC Australia; ^5^ Department of Clinical Pathology, Melbourne Medical School The University of Melbourne Parkville VIC Australia; ^6^ Institute for Computational Cancer Biology (ICCB), Center for Integrated Oncology (CIO), Cancer Research Center Cologne Essen (CCCE) Faculty of Medicine and University Hospital Cologne, University of Cologne Cologne Germany; ^7^ BIFOLD – Berlin Institute for the Foundations of Learning and Data Berlin Germany; ^8^ Berlin Institute for Medical Systems Biology (BIMSB) Max Delbrück Center for Molecular Medicine in the Helmholtz Association (MDC) Berlin Germany; ^9^ Nottingham Breast Cancer Research Centre, Academic Unit of Translational Medical Sciences, School of Medicine University of Nottingham and Department of Histopathology, Nottingham University Hospitals NHS Trust, City Hospital Nottingham UK; ^10^ Pathology Department, Faculty of Medicine Menoufia University Shibin Al Kawm Egypt; ^11^ Pathology Queensland Royal Brisbane and Women's Hospital Brisbane QLD Australia; ^12^ Centre for Clinical Research The University of Queensland Brisbane QLD Australia; ^13^ Pathology Department Hamad Medical Corporation Doha Qatar; ^14^ Present address: Lab of Systems Pharmacology Harvard Medical School Boston MA USA; ^15^ Present address: Department of Genetics The University of Texas, MD Anderson Cancer Center Houston TX USA

**Keywords:** ductal carcinoma *in situ*, recurrence, clonality, breast neoplasm, whole exome sequencing, phylogenetic analysis

## Abstract

Development of ipsilateral breast carcinoma following a diagnosis of breast ductal carcinoma *in situ* (DCIS) has been assumed to represent recurrence of the primary tumour. However, this may not always be the case, and it is important to determine how often such recurrences represent new tumours. Ipsilateral primary–recurrence pairs (*n* = 78) were sequenced to test their clonal relatedness. Shared genetic events were identified from whole exome sequencing (*n* = 54 pairs) using haplotype‐specific copy number and phylogenetic analysis. The remaining pairs were sequenced using a targeted panel or low‐coverage whole genome sequencing. We included 32 non‐recurrent DCIS to compare recurrent and non‐recurrent disease. We found that 7% of DCIS recurrences were non‐clonal by whole exome sequencing, indicative of a new breast carcinoma. Lower resolution methods detected a higher non‐clonality rate (29%). By comparing primary DCIS with their recurrence, we found that the evolution of DCIS to invasive disease was associated with increased ploidy and copy number events. *TP53* mutations were enriched in DCIS with clonal recurrence compared with non‐recurrent DCIS. Our results verify that *de novo* ‘recurrent tumours’ of independent origin occur in patients who may be at high risk. © 2025 The Author(s). *The Journal of Pathology* published by John Wiley & Sons Ltd on behalf of The Pathological Society of Great Britain and Ireland.

## Introduction

Ductal carcinoma *in situ* (DCIS) is a pre‐invasive breast lesion and is a known non‐obligate precursor of invasive breast cancer (IBC). Since up to 25% of DCIS may recur as either DCIS or IBC [1], DCIS is treated with surgery, with or without radiotherapy, and/or systemic endocrine therapy. It has long been recognised that this approach overtreats many women due to a lack of accurate prognostic markers of recurrence [2, 3]. Treatment options remain the same for patients with a recurrent tumour: further surgery with or without radiotherapy for recurrences of DCIS and additional systemic therapies for IBC, if indicated.

There has been an assumption that ipsilateral breast carcinomas that develop after a diagnosis of DCIS are genetically related (i.e. clonal) to the primary tumour (true recurrences), but this may not always be the case. Using somatic copy number alterations (CNAs) derived from SNP arrays, we previously showed that two out of eight recurrences were non‐clonal (i.e. new primaries) [4]. Others have reported that 18% of IBC and 9% of DCIS recurrences are non‐clonal [5]. Knowing the true recurrence rate not only is important to guide patient management but is also critical in designing studies that aim to identify DCIS biomarkers of recurrence. If the rate of non‐clonal recurrences is high, then tumour‐intrinsic‐based biomarker discovery is compromised and strategies will be needed to identify those at risk of new primaries.

The aim of this study was to determine the clonal relatedness and evolutionary trajectory of primary DCIS and subsequent carcinoma pairs in a large cohort of patients. For simplicity, we will refer to all such second events as ‘recurrences’, even when non‐clonal. We performed genetic analysis of both non‐recurrent DCIS and primary–recurrence tumour pairs with whole exome sequencing (WES), low‐coverage whole genome sequencing (LCWGS), or a targeted sequencing panel to provide an insight into the molecular features of recurrences.

## Materials and methods

### Ethics approval

This study was conducted under ethical approval from the Peter MacCallum Cancer Centre (PMCC) (HREC#16/PMCC/122), Melbourne Health (HREC#2008.219), St Vincent's Hospital (HREC#022‐19), and the North West–Greater Manchester Central Research Ethics Committee (15/NW/0685).

### Patient samples

Primary DCIS and recurrent DCIS/IBC (total *n* = 82, including *n* = 78 ipsilateral and *n* = 4 contralateral) as well as non‐recurrent DCIS (*n* = 32) cases were identified through hospital databases from the Royal Melbourne Hospital (RMH), Nottingham City Hospital (UK), the LifePool cohort, and PMCC. Patients were non‐recurrent when there was no record of a second DCIS/IBC within 7 years of the initial DCIS diagnoses after breast‐conserving surgery (i.e. no mastectomy; median follow‐up 8.5 years, range 7–23 years). In contrast, recurrent patients were diagnosed with either DCIS or IBC more than 1 year following their initial DCIS diagnosis regardless of the surgery type. We performed genomic analysis on 82 pairs of DCIS with recurrence and 32 DCIS without recurrence. Out of 82 pairs, four pairs were contralateral, anticipated, and confirmed to be non‐clonal controls, and therefore not included in summarised information or comparisons. All of the ipsilateral recurrent and non‐recurrent cases are described in supplementary material, Table S1.

### DNA extraction and sequencing

**Figure 1:**
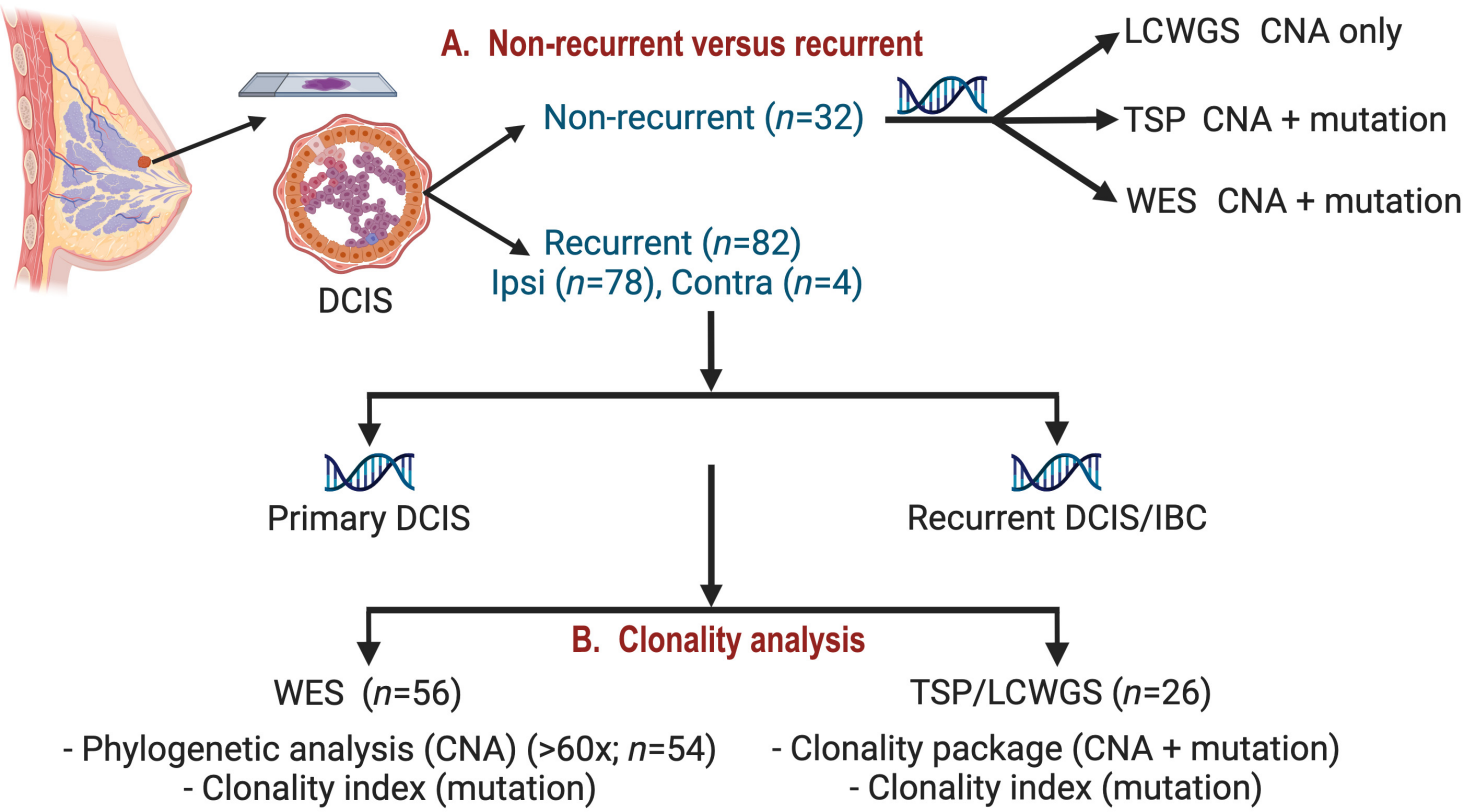
Experimental design. Non‐recurrent DCIS (*n* = 32) and 82 pairs of matched primary DCIS–recurrent tumours were microdissected followed by DNA extraction. Four contralateral pairs were tested as non‐clonal controls. All other analyses focused on either primary–recurrence pairs (*n* = 78) or non‐recurrent DCIS (*n* = 32). Depending on DNA availability and sequencing technology, clonality analyses were performed by the Clonality package, clonality index, and/or by investigating the evolutionary history by phylogenetic analysis. For 56 WES pairs, 54 had sufficient depth and were considered for phylogenetic analysis, while the remaining two pairs’ clonality was assessed by other methods, similar to low‐depth sequencing. CNA, copy number alteration; TSP, targeted sequencing panel; LCWGS, low‐coverage whole genome sequencing; WES, whole exome sequencing. This Figure is created with BioRender.com.

All cases were microdissected from haematoxylin‐stained sections (range 4–20 sections) by manual microdissection to achieve > 50% tumour tissue purity. DNA was extracted from formalin‐fixed, paraffin‐embedded (FFPE) tissue using the FFPE AllPrep Kit (QIAGEN, Germantown, MD, USA). Depending on the success and timing of DNA extraction (Figure 1), cases were analysed by a targeted sequencing panel (*n* = 46, samples pre‐2020), WES (*n* = 67), or LCWGS. For WES, DNA was processed by the Australian Genome Research Facility (AGRF) using the Twist Bioscience Human Comprehensive Exome v1 or v2 (Twist Bioscience HQ, South San Francisco, CA, USA) according to the Twist Target Enrichment Standard Hybridization Protocol (https://www.twistbioscience.com/resources/protocol/twist-target-enrichment-standard-hybridization-v2-protocol). Sequencing was performed on the Illumina NovaSeq 6000 (Illumina, San Diego, CA, USA) with 150 bp paired‐end reads for a median depth of 101.29×, range 20.39–247×. For whole genome sequencing (WGS), libraries for four paired samples with matched stromal DNA were prepared, processed, and sequenced by AGRF using the IDT xGen Kit (Integrated DNA Technologies, San Diego, CA, USA) following the manufacturer's protocol, and aiming to achieve a depth of 60× for tumour DNA and 30× for matched normal DNA.

Targeted sequencing was performed on a panel of known IBC driver genes [6] (see supplementary material, Table S2 for gene list). Library preparation was performed by the Molecular Genomics Core, PMCC, mostly with an input of at least 100 ng of DNA using the KAPA HyperPrep Kit [Roche Diagnostics (Switzerland) Ltd, Rotkreuz, Switzerland] and the Agilent SureSelect XT hybridisation capture system (Agilent, Santa Clara, CA, USA) as previously described for this panel [7, 8]. Sequencing of target‐enriched DNA libraries was performed using the Illumina NextSeq 500 (Illumina), generating 75 bp paired‐end reads. For LCWGS, a low DNA input library preparation protocol was used for samples with less than 30 ng of DNA, using the NEBNext® Ultra™ II DNA Library Prep Kit (New England BioLabs Inc., Ipswich, MA, USA; Cat. No. E7645S/L) as previously described [8, 9, 10]. The Illumina NextSeq 500 paired‐end 75 bp run achieved sequencing depths of approximately 2×.

A subset of cases had distant stroma available for DNA extraction and each sequencing run had at least one stromal DNA sample as a control.

### Data analysis

Sequence reads were aligned to the hg19 reference genome using BWA v0.7.17 [11]. Duplicate read removal, local realignment, and variant calling were performed as described in Supplementary materials and methods. Called variants were additionally annotated using the Ensembl Variant Effect Predictor (VEP), release 78 [12] and filtered for high confidence variants, with high stringency population filtering to enrich for somatic variants.

Both off‐target and on‐target sequencing reads were used to generate genome‐wide copy number data using PureCN v2 [13]. Normal DNA samples (*n* = 24) were pooled and used as a baseline for PureCN for most WES cases (Exome v1), except for 12 primary–recurrence pairs for which only one normal DNA was used (Exome v2). LCWGS was aligned and used for copy number analysis using ControlFREEC (v6.7) [14] with 50 kb windows as previously described [8, 9, 10, 15]. Fraction of genome altered (FGA) was then calculated as previously described [4].

CNA profiles, purity, and ploidy status from solution 1 of PureCN data from WES and WGS were used to generate haplotype‐specific copy number changes by multi‐sample phasing using Refphase (https://bitbucket.org/schwarzlab/refphase/src/master/) as previously described [16]. Samples with insufficient depth and low quality failed to phase and were excluded (DCIS00191, DCIS00401). Phylogeny reconstruction and ancestral genomes between primary–recurrence pairs were inferred by Minimum Event Distance for Intra‐tumour Copy‐number Comparisons‐2 (MEDICC2) [17]. Clonality was also evaluated for all cases using a statistically based clonality analysis with the Clonality R package (version 3.6), incorporating total CNAs, and mutations, when available, for both tumours in the pair [18, 19]. An additional estimation for clonal relatedness was performed as described by Schultheis *et al* [20], called clonality index (CI) and CI2. These tools estimate clonality based on the number of shared mutations and their frequency in a control data set. Further details are provided in Supplementary materials and methods and supplementary material, Figures S1–S4. Manual checking of copy number, Refphase, and MEDICC2 plots was performed to verify the clonality predictions.

### Histology and tumour‐infiltrating lymphocytes (TILs)

Estrogen receptor (ER) was determined as reported on the pathology record at the time of diagnosis or was performed as previously described [21]. HER2 amplification status was determined from copy number data [4]. TILs of non‐recurrent and recurrent cases were available from pathological review at the time of diagnosis or were assessed later for this study by breast pathologists (JMP or NR) using the method by Pruneri *et al* [22] for DCIS.

### TP53 immunohistochemistry (IHC)

Tissue microarrays (TMAs) were obtained from the Department of Histopathology, Nottingham University Hospitals NHS Trust, Nottingham, UK [21] and Centre for Clinical Research, the University of Queensland, Brisbane, Queensland, Australia. TP53 IHC was performed by the Peter MacCallum Department of Anatomical Pathology using the standard clinical assay (clone DO7) [6]. Scoring was performed blinded to mutation status and outcome, and required at least 50 tumour cells to be present. Cases were called normal if there was heterogeneous staining in < 50% of cells and no contiguous areas of staining. Cases were abnormal if they carried one of three mutation‐related patterns [23]. Overexpression (OE) included strong nuclear staining in > 5% contiguous cells and required contiguously stained cells across at least 50% of a duct containing at least 20 cells. Complete absence (CA) was no staining in any tumour cells, but positive staining in some stromal or lymphocyte cells. Cytosolic staining (CY) was diffuse cytoplasmic staining in cells in the same proportions as for OE. Ten cases on the TMA that were included in the TP53 survival analysis were also in the discovery cohort.

### Statistical analyses and data visualisation

GraphPad Prism version 9.2.0 (GraphPad Software Inc., San Diego, CA, USA), R version 4.3.2 [24], and RStudio (version 2023.09.1; Posit Software, Boston, MA, USA) were used to generate graphs and perform statistical tests and analyses. *p* < 0.05 was considered significant unless stated otherwise and all tests were two‐tailed. The association of *TP53* mutation with recurrence was tested using a multivariable logistic regression model in R (*stats::glm*) adjusting for grade, ER status, and radiotherapy. Univariable and multivariable Cox proportional hazards models were performed on the IHC cohort to identify clinical and molecular characteristics associated with patients’ recurrence risk (*survival::coxph* version 3.5–7) [25]. The proportional hazard assumption was assessed using the test based on Schoenfeld residuals and graphical methods [26]. More details are provided in Supplementary materials and methods.

## Results

### Clinicopathological characteristics of the cohort

We performed genomic analysis on 78 pairs of DCIS with ipsilateral recurrence and 32 DCIS without recurrence (Figure 1 and supplementary material, Table S1). When we compared the recurrent and non‐recurrent cohorts, there was no significant difference in patients’ age at the initial diagnosis of DCIS, ER/HER2 status of primary DCIS, or the size of primary DCIS (Table 1 and supplementary material, Figure S5). DCIS with recurrence were more likely to be high grade and not treated with radiotherapy (Table 1 and supplementary material, Figure S6 and Table S3).

**Table 1:** Cohort characteristics.

Variable	Non‐recurrent	Recurrent	*p* value^†^	Clonal recurrent
	*n* = 32*	*n* = 78*		*n* = 67
Age (years)	61.3 (± 7.5)	60.4 (± 9.4)	0.5	60.6 (± 8.9)
Unknown	0	3		2
Radiotherapy	11/17 (65%)	13/71 (18%)	<0.001	12/62 (19%)
Unknown	15	7		5
Grade			0.042	
High	14/32 (44%)	51/78 (65%)		45/67 (67%)
Intermediate	14/32 (44%)	16/78 (21%)		13/67 (19%)
Low	4/32 (13%)	11/78 (14%)		9/67 (13%)
Size (mm)	16.1 (± 12.2)	18.8 (± 17.1)	0.6	20.4 (± 17.8)
Unknown	0	2		1
ER			0.3	
Negative	4/32 (12.5%)	17/75 (23%)		16/66 (24%)
Positive	28/32 (87.5%)	58/75 (77%)		50/66 (76%)
Unknown	0	3		1
HER2			0.8	
Negative	24/32 (75%)	55/78 (71%)		46/67 (69%)
Positive	8/32 (25%)	23/78 (29%)		21/67 (31%)
Tumour lymphocytes	11.3 (± 8.4)	14.5 (± 17.3)	0.8	15.1 (± 17.6)
Unknown	5	26		18

* Mean (SD); *n*/*n* (%).

^†^ Wilcoxon rank‐sum test; Pearson's *χ* ^2^ test; non‐recurrent versus recurrent.

### Phylogenetic analysis and somatic mutations identified independent ipsilateral recurrent tumours

**Figure 2:**
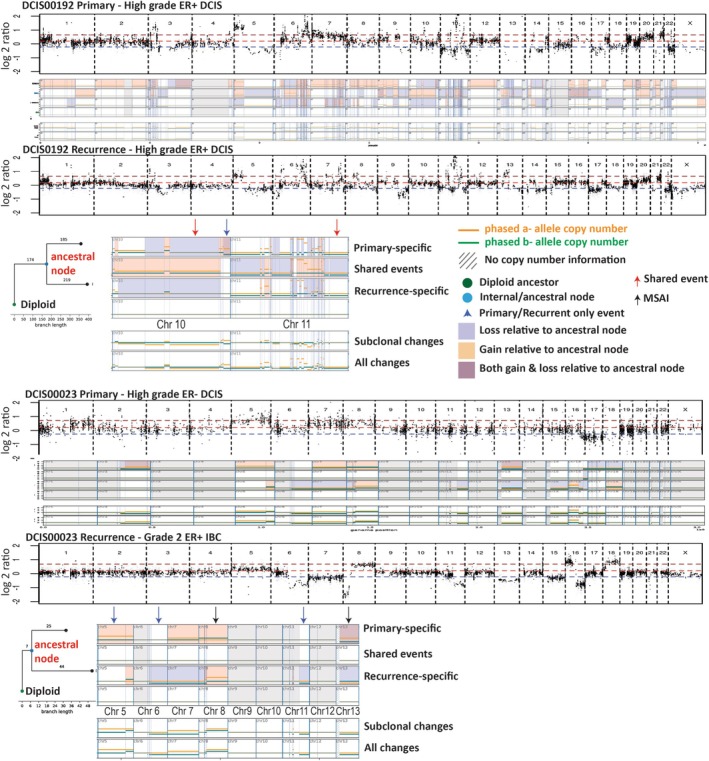
Example of a clonal pair and a non‐clonal pair sequenced by high‐depth whole exome sequencing (WES). These profiles were generated by Refphase (log_2_ plots) and MEDICC2 (summary events) based on haplotype‐specific copy number profiles. Chromosomes 10 and 11 are magnified for visualisation. First, in case DCIS00192, the phylogenetic tree suggests that there were 174 clonal segments identified in the ancestral genome (internal node; red arrows indicate examples), indicating truncal events in the primary DCIS and recurrent IBC. The recurrent tumour had 219 divergent CNA segments and the primary had 185, indicating subclonal events for both tumours that occurred after the clonal events (examples: blue arrows). For case DCIS00023, a few small segments on Chr16 were detected as truncal events (truncal segments *n* = 7), but these did not share convincing breakpoints and this is a common deletion in breast cancer. Lack of other shared CNAs and mutations suggested that this pair was non‐clonal, which was confirmed by whole genome sequencing. An example of mirrored subclonal allele imbalance (MSAI) is shown on chromosomes 8 and 13 (black arrow), suggesting loss of different alleles on the same CNA arms (i.e. parallel evolution).

We obtained high‐depth WES data for 54/78 paired primary–recurrence cases and undertook phylogenetic analysis using Refphase and MEDICC2. Refphase incorporates allelic information when assessing CN events. MEDICC2 is based on the minimum event distance between all genomes using neighbour joining taking into account whole genome doubling (WGD) events [17]. As opposed to looking at any chromosomal event as a single event, such an analysis may reveal the evolutionary history of CNAs. Supplementary material, Figure S7 shows an example of a case that would likely be called non‐clonal by examination of CNAs but was classified as clonal (i.e. shared ancestral genomes) by investigating their evolutionary history. Of these 54 pairs, 49/54 (91%) were classified as clonal (i.e. shared an ancestral genome; see Figure 2).

**Figure 3:**
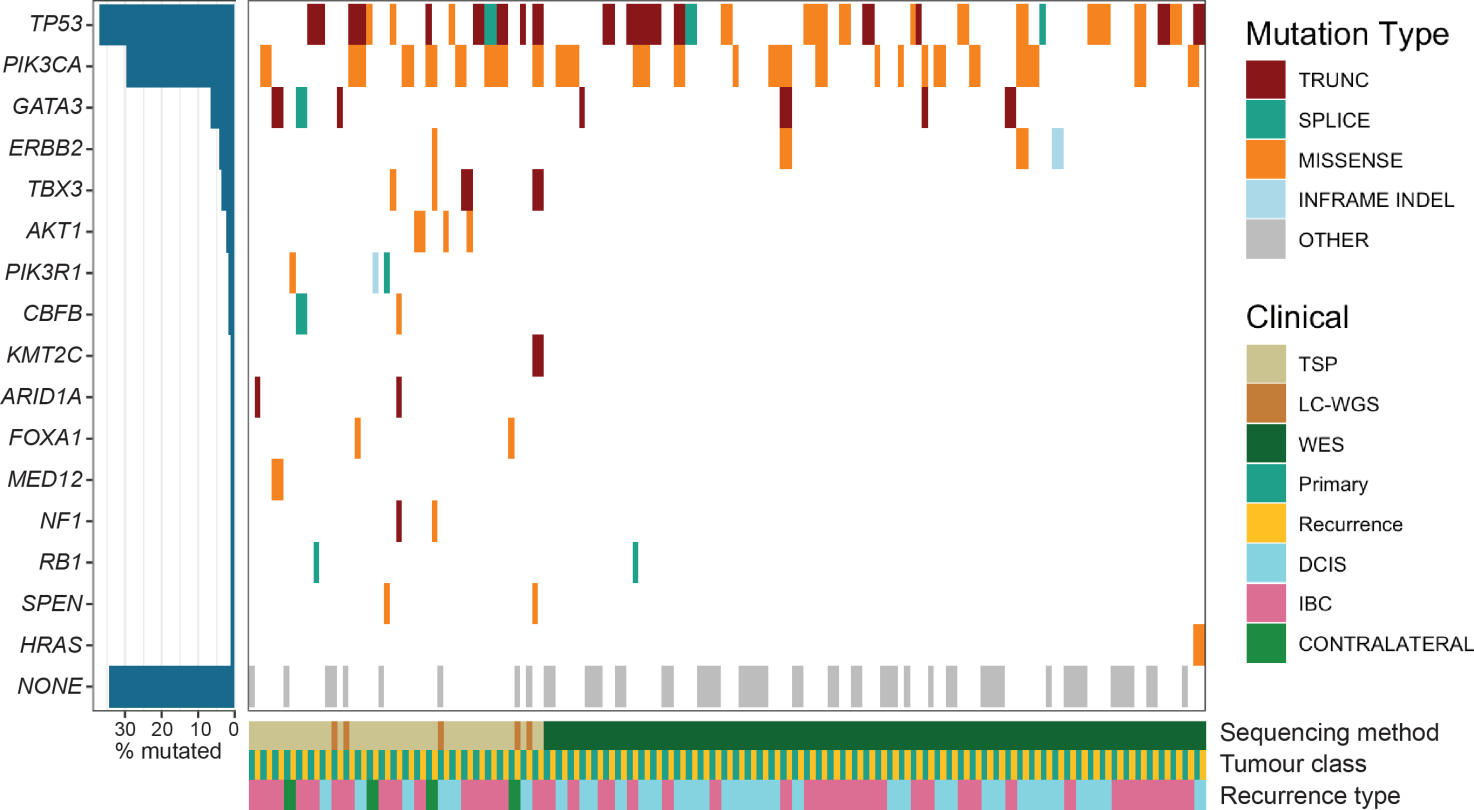
OncoPrint profile for somatic mutations of recurrent cases. For each paired case, both primary (teal) and recurrent (gold) tumours are shown. Different types of mutation are indicated for each gene, with the frequency of total mutation observed in a particular gene given. Grey boxes indicate a tumour with no mutations in the gene list indicated on the left, which is filtered for genes frequently mutated in breast cancer. The recurrence and sequencing method are also shown. This profile was generated using GenVisR v1.36 [36].

When we investigated somatic mutations (Figure 3), one out of six non‐clonal pairs became clonal due to a shared somatic *PIK3CA* p.E542K mutation (case DCIS00009) (supplementary material, Table S4). Four of the remaining five pairs had enough DNA for whole genome sequencing with matched normal stromal DNA to check their clonal relatedness. Three were confirmed as non‐clonal (supplementary material, Figure S8). However, DCIS00506 contained a small deletion at the end of chromosome 16 that, along with 1q gain and a shared *PIK3CA* mutation (p.H1047R, which alone was insufficient for the case to be called clonal), suggested a clonal origin (supplementary material, Figure S9). Taken together, high‐depth sequencing identified 4/54 cases as non‐clonal (7.4%), of which three were invasive breast cancer.

We also evaluated clonality in 24 ipsilateral and four contralateral cases analysed by lower resolution methods including targeted sequencing, LCWGS, and low‐coverage WES using two statistical methods [19, 20] and manual verification of CNAs (Supplementary materials and methods; supplementary results). All contralateral cases were non‐clonal. The rate of calling non‐clonality in ipsilateral cases was significantly higher using these lower resolution methods (7/24, 29.2% compared with 7.4% for WES/WGS; *p* = 0.03, Fisher's exact test). Three were invasive and four were DCIS recurrences. Clonality detection was not strongly affected by the purity of the tumour DNA, which was not significantly different between primary DCIS with non‐clonal and those with clonal recurrence (*t*‐test *p* = 0.32) or between the non‐clonal and clonal recurrences (*t*‐test *p* = 0.98).

### Phylogenetic analysis showed the presence of ongoing chromosomal instability in DCIS and IBC recurrences

**Figure 4:**
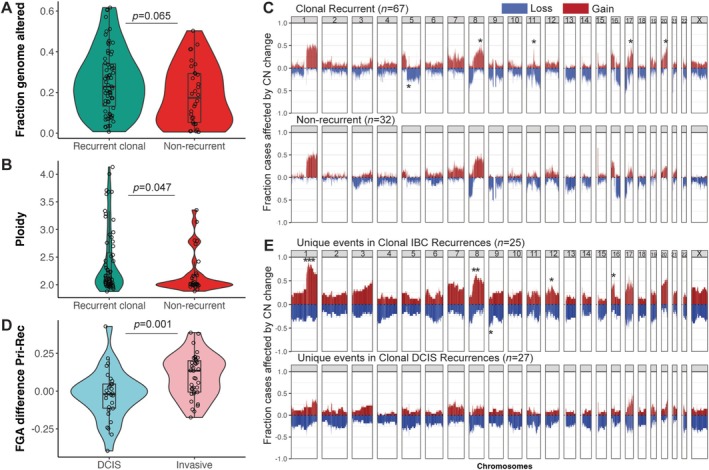
Comparison between the non‐recurrent and recurrent cohorts. (A) Fraction of genome altered (FGA) by copy number. (B) Ploidy. The *p* value is the comparison between cases with a clonal recurrence and non‐recurrent cases by a Wilcoxon rank‐sum test. (C) Genome‐wide copy number frequency of non‐recurrent cases (*n* = 32) and cases with clonal recurrence (*n* = 67). The chromosome number (1–X) is given at the top. Blue indicates loss and red indicates gain. (D) Difference in FGA between the primary and the recurrence. *p* value is the comparison between cases with a clonal DCIS versus clonal invasive recurrence by a Wilcoxon *t*‐test. (E) Copy number frequency plot for regions specifically altered in the recurrence tumours (derived from WES samples analysed by MEDICC2). This includes regions where a CNA was already present in the truncal clone and has been further changed in the recurrence sample. Note that when different alleles have been affected by different copy number events (e.g. gain of allele ‘A’ and loss of allele ‘B’), both will be represented in the frequency plot. The chromosome number (1–X) is given at the top. Blue indicates gain and red indicates loss. *p < 0.05, **p < 0.01, ***p < 0.001, Fisher's exact tests.

We next investigated the evolutionary history of the clonal pairs. Overall, the proportion of the genome affected by CNAs was higher in the clonal recurrences (median 30.8%) than in the index lesion (median 22.9%, *p* = 0.027, paired *t*‐test; supplementary material, Figure S10). This was primarily driven by an increase in FGA in the IBC recurrences (median increase of 13%, *p* = 0.0001, paired *t*‐test; Figure 4D). MEDICC2 analyses showed that all DCIS with clonal recurrence harboured subclonal events (range 30%–80%, median 55.7%), indicating ongoing chromosomal instability (supplementary material, Figure S11). There was no significant difference in terms of the proportion of clonal CNAs of primaries whether they recurred as DCIS or IBC (*p* = 0.49, Wilcoxon test; supplementary material, Figure S11). The clonal diversification did not seem to be dependent on grade, ER or HER2 status, or *TP53* mutation status of the primary DCIS (*p* > 0.05 for all, Wilcoxon or Kruskal–Wallis test). There was a trend for invasive recurrences to have a longer time to recurrence (median 4.5 years compared with 2.8 years for DCIS recurrences, *p* = 0.05, Wilcoxon test; supplementary material, Figure S11).

MEDICC2 revealed the presence of WGD events. No non‐recurrent DCIS (0/10) had WGD, but 6/52 DCIS with clonal recurrence (11%, *p* = 0.58, Fisher's exact test; supplementary material, Figure S12) and eight clonal recurrences (15%) showed WGD events. Genome‐doubling events were truncal for three pairs, all with IBC recurrence (supplementary material, Figure S13). All cases also had multiple subsequent gains and losses unique to the primary and the recurrence. When considering ploidy as an alternative measure of aneuploidy, overall there was no significant difference between primary DCIS and their clonal recurrences (*p* = 0.23, paired *t*‐test), but invasive recurrences had higher ploidy than their primary DCIS (*p* = 0.03, paired *t*‐test), similar to what we observed with the FGA (supplementary material, Figure S11).

We further investigated the development of CNAs in invasive recurrences to see whether any specific events were enriched. Using the segments flagged as being unique to the recurrence tumours by MEDICC2, we compared the frequency of new events in DCIS and invasive recurrences. As expected, invasive recurrences had globally higher levels of CNAs compared with DCIS recurrences, and showed significant increases in gains at 1q, 8q, 12q, and 16p, and losses at 9p (Figure 4E).

Clonal diversification was also seen at the mutation level. Thirty‐seven clonally related cases shared somatic driver mutations, most commonly in *TP53* (*n* = 24) and *PIK3CA* (*n* = 16). However, 11/63 cases carried variants present only in the primary or the recurrence sample and 15 carried no clearly somatic variants in either sample. There was no significant difference in the presence of matching variants between DCIS (15/30) and IBC (22/33) recurrences (*p* = 0.28, *χ* ^2^ test). Intriguingly, we found evidence of parallel and convergent evolution, whereby the same gene was mutated in the primary and the recurrence but at a different site. Two clonal cases carried divergent *TP53* mutations and one had different *PIK3CA* mutations (parallel evolution), while two non‐clonal cases had distinct *TBX3* and *PIK3CA* mutations, respectively (convergent evolution).

Taken together, our data show the complex genetic evolution of DCIS and its recurrences, mostly suggesting diverse evolutionary trajectories across cases and with a tendency for true invasive recurrences to be more different to their primary DCIS at a copy number level.

### Clonal cases showed a significant enrichment of specific CNAs and *TP53* mutation

With the aim of predicting the likelihood of a recurrence after an initial diagnosis of DCIS, we compared cases with recurrence with 32 non‐recurrent cases analysed with either the targeted sequencing panel (*n* = 18), WES (*n* = 11), or LCWGS (*n* = 3). Overall, the estimated ploidy was higher in DCIS with clonal recurrences than in non‐recurrent DCIS (*p* = 0.047, Wilcoxon rank‐sum test). Total fraction of the genome affected by CNA for the DCIS with clonal recurrences was somewhat but not statistically significantly higher than non‐recurrent cases (*p* = 0.065, Wilcoxon rank‐sum test, Figure 4). CNAs such as gain of *CCND1* (on 11q) and *ZNF217* (on 20q) genes, as well as copy number changes on 17q and 5q, were more evident in DCIS with clonal recurrences than in non‐recurrence cases (Figure 4C and supplementary material, Figures S14 and S15). However, the change in 5q may be influenced by a greater presence of ER‐negative cases in clonal recurrence cases as this CNA was significantly different between ER‐positive clonal and ER‐negative clonal cases (12/44 ER+ versus 12/15 ER−; *p* = 0.0006, Fisher's exact test). *PIK3CA* mutations were slightly more common in non‐recurrent DCIS than in DCIS with clonal recurrences but this was not statistically significantly different (*p* = 0.068, Fisher's exact test; supplementary material, Figure S16). *TP53* mutations (but not copy number loss) were significantly enriched in DCIS with clonal recurrences compared with non‐recurrent DCIS (*p* = 0.009, Fisher's exact test; supplementary material, Figure S16). We evaluated whether this was affected by including the potentially confounding variables grade, ER status, and radiotherapy in a multivariable logistic regression model (supplementary material, Tables S7 and S8). *TP53* mutation had an odds ratio of 6.99 (95% CI 1.11–74.4) in this model.

To evaluate *TP53* mutations using an orthogonal method, we performed TP53 IHC (Supplementary materials and methods; supplementary results; supplementary material, Figures S16, S17 and Tables S5 and S6). Protein staining was concordant with the mutation status in 31/35 (89%) cases with both data types (supplementary material, Figure S18). Abnormal TP53 status by IHC in an extended cohort was significantly associated with ipsilateral recurrence in univariable and multivariable analyses (supplementary material, Figures S16, S18 and Table S9). However, when removing the ten cases that overlapped with the genetic cohort (9/10 with recurrence), these associations were no longer statistically significant (supplementary material, Figure S18 and Table S9).

## Discussion

This study challenges the previous assumption that later ipsilateral breast carcinomas after a prior DCIS are directly related (i.e. true recurrence with clonal somatic genetic events). Clonality implies that the recurrence has arisen from tumour cells that survived treatment. Using haplotype‐specific copy number profiles, we show that *de novo* breast carcinomas occur in at least 7% of patients and that clonality status cannot be accurately predicted by shared grade or receptor status. Lips *et al* recently showed that 18% of IBC recurrences in a large cohort were unrelated to the primary DCIS [5]. This higher rate could be explained by their analysis of most paired samples by LCWGS and some targeted sequencing in keeping with our observed decrease in the non‐clonal proportion of cases when moving from lower (29%) to higher (7%) resolution sequencing. In comparing the two studies, it is worth noting that due to the small sample sizes and analytical limitations, the confidence in each observed rate is low. Regardless, Lips *et al* did confirm two cases as non‐clonal using single‐cell DNA sequencing, reflecting independent lineages. They also were unable to identify any characteristics that could predict clonal versus non‐clonal recurrence.

Notably, all but two of the cases could be resolved without somatic point mutation data. It remains possible that the apparently independent recurrences were clonally related to the primary DCIS at a level prior to any detectable driving somatic genetic events. The tumour could have been initiated through epigenetic alterations, and the pair could share passenger somatic variants from an early clonal expansion that we could not detect because of the lack of matching germline DNA for most cases. Because of this limitation, the mutational landscape remained understudied in our cohort, and in‐depth phylogenetic analysis such as PyClone could not be carried out. Similarly, the combination of few variants and FFPE‐derived DNA made it inadvisable to attempt to estimate mutational signatures.

Further limitations include extracting DNA from only one block per sample. Therefore, we would not have identified any independent clones present in a different region of the tumour, i.e. polyclonal DCIS [27, 28], although this is unlikely as all non‐clonal primary DCIS were small (< 17 mm; supplementary material, Table S3). Another possibility would be an occult ‘second’ primary tumour elsewhere in the same breast at the time of the primary DCIS diagnosis (multi‐focality) that was not detected by mammogram [29]. Additional concerning foci have been reported on pre‐operative magnetic resonance imaging (MRI) but missed by conventional mammogram. In addition, the precise locations of recurrent tumours were unavailable for most cases in our cohort and so the distance between them and the primary DCIS could not be investigated.

As well as increasing our clinical understanding around DCIS recurrence, we were also interested in whether the paired analysis could inform us of the genetic events underpinning the DCIS–IBC transition. This was challenging due to high inter‐tumour heterogeneity, and some clonal primary–recurrent tumour pairs showing strong clonal diversification reflecting ongoing chromosomal instability. These issues could be resolved in the future through multi‐region sampling or, in an era of spatial biology, profiling cases by interrogating the whole tumour at very high resolution, which may indicate how subclonal populations are spatially resolved. We also showed the presence of parallel evolution in DCIS and their clonal DCIS/IBC recurrences, an event that has been shown in IBC [16], which might indicate a strong microenvironmental selection pressure. Despite this complexity, we did find that invasive recurrences had higher levels of CNAs, including enrichment for some chromosomal loci. Previous studies examining the evolution of DCIS to IBC have commonly analysed synchronous tumours, which are limited by retrograde cancerisation of ducts by IBC. For example, Pareja *et al* performed phylogenetic analysis of WES data in a small cohort of DCIS and DCIS synchronous with IBC [30]. They and others showed that DCIS co‐existing with IBC had similar genetic events including CNAs but also showed evidence of WGD events in DCIS and clonal diversification [30, 31, 32]. However, this study lacked recurrent samples and so could not capture the biology of true DCIS recurrences occurring later in time. We have now shown similar truncal WGD and clonal diversity in our time‐separated lesions and were also able to identify CN events enriched in the IBC recurrences. In our previous study of DCIS synchronous with IBC, we similarly noted an increased amplitude or presence of CN gain in IBC regions on chromosomes 8q and 16p [33]. Recurrence‐specific gains on 1q, and to a lesser (non‐significant) extent on 8q, 12q, and 16p, were also noted in the study by Lips *et al*. The increases in specific CN events in invasive recurrences reflect the overall enrichment for some of these regions in primary DCIS with recurrences. Some cases may have had these events present in a clone in the primary DCIS that was not sampled by our analysis, but these differences may also reflect continued CN evolution during the acquisition of an invasive phenotype.

The finding that at least 7% of so‐called recurrences are actually *de novo* new primary tumours supports the idea that some patients might have a high‐risk microenvironment and/or genetic predisposition for new tumour development. Our findings are likely to significantly affect patient management as *de novo* tumours will be treated differently from recurrent tumours, with benefit then from genetic testing or preventive therapy in the same manner as someone with a strong family history, known genetic predisposition, or bilateral breast cancer [34]. Indeed, *BRCA* mutation carriers have a higher risk of ipsilateral new primary tumours after IBC treated by breast‐conserving surgery compared with non‐carriers, particularly in the longer term. We detected probable germline pathogenic/likely pathogenic variants in one non‐clonal case (in *ATM*) and two clonal recurrence cases (in *BRCA2* and *PALB2*). Unfortunately, the low number of cases and limited germline data precluded establishing any increase in the prevalence of germline variants in non‐clonal recurrence cases.

We assessed the prognostic and potentially predictive association of TP53 abnormality using IHC in a dataset that included cases from the discovery cohort. Along with the enrichment of key CNAs, our findings suggest these molecular biomarkers could be used to personalise treatment, although we acknowledge the potential for bias in our non‐matched case–control study and the limitation in sample size. Our clonality study raises two important issues in developing such a biomarker: first, will the discovery and validation of tumour‐intrinsic biomarkers be compromised by including non‐clonal cases? This depends on the true rate of non‐clonal recurrences. If relatively uncommon as with this study (7%), our data suggest that for strong biomarkers it may have a limited impact. The odds ratio for *TP53* mutations in the discovery cohort dropped from 4.49 (95% CI 1.33–19.8) to 4.2 (95% CI 1.27–18.3) when all cases were included. However, if more frequent as observed in other studies, it may have a profound effect especially for less powerful biomarkers. The second implication is whether the application of a tumour‐intrinsic DCIS biomarker will lead to undertreatment of the patients who are at risk of a *de novo* tumour. These patients could carry a ‘low‐risk’ tumour molecular profile in their primary DCIS that might lead to a less aggressive treatment course. This impact could be alleviated by the development of complementary tumour microenvironmental biomarkers [35] as well as the identification of germline risk factors through mainstreaming of pathogenic variant detection in women with DCIS. Although elements of the tumour microenvironment have previously been associated with recurrence risk, such as stromal lymphocytes and collagen structures, no studies have as yet evaluated whether these are associated with clonal recurrences or *de novo* primaries.

## Author contributions statement

KLG, GBM, SBF and TK conceived and designed the study. GBM, EAR, SBF, ARG, IGC, LD, SIJ, SL and MC provided access to clinical samples. JMP, EH, SIJ and NR performed the pathological review. SM, SH, HS, DL, HN, OC, MP, LT, TS, AL, RL and DJB performed experiments. NR and J‐MP performed TIL counts. Bioinformatics support, statistical support and data analysis were provided by MZ, RiL, TK, SL and MWJ. Phylogenetic analysis was conducted by TK, RFS and TLK. Acquisition of data, analysis and interpretation of data were performed by TK and KLG. DJB, IMM, DL, SH, EH and LD were responsible for identification of cases and performing additional experiments. TK and KLG drafted the manuscript. KLG was responsible for overall study supervision and obtaining ethics approval. All authors read and approved the final manuscript.

## Supporting information

Supplementary materials and methods
**Figure S1**. Log ratio profiles generated by the Clonality package (R version 3.6.2) comparing a paired tumour based on total SCNA
**Figure S2**. Discrepancy between the Clonality package and manual inspection for a non‐clonal case
**Figure S3**. Example of three non‐clonal pairs
**Figure S4**. Refphase profile of LP005
**Figure S5**. Correlation of various clinico‐pathological and genetic features with recurrence
**Figure S6**. Correlation of time to recurrence and radiotherapy with recurrence
**Figure S7A**. Illustration of the reasoning behind choosing phylogenetic analysis (with higher‐depth WES) over manual inspection of somatic copy number alterations (SCNAs) and breakpoints
**Figure S7B**. Example of a clonal pair and a non‐clonal pair sequenced by whole exome sequencing (WES)
**Figure S8**. Refphase profile for DCIS0276 WES (left) and WGS (right)
**Figure S9**. Refphase profile for DCIS0506 WES (left) and WGS (right)
**Figure S10**. Comparisons between primary and recurrent tumours
**Figure S11**. Differences by recurrence histology
**Figure S12**. Genomic features by recurrence
**Figure S13**. Examples of clonal pairs with a truncal WGD event and a subclonal WGD event
**Figure S14**. CNA frequency for selected chromosomes
**Figure S15**. CNA frequency for chromosome 8 highlighting *RAD21* (significantly different CN gain frequency between non‐recurrent and non‐clonal recurrence) and *MYC* (not different)
**Figure S16**. Association of mutations with recurrence
**Figure S17**. TP53 immunohistochemistry
**Figure S18**. TP53 staining association with recurrence with all cases (left) and with cases also present in the genetic cohort (*n* = 10) removed (right)
**Table S1**. Sample information
**Table S2**. List of genes on targeted sequencing panel
**Table S3**. Differences by recurrence clonality status
**Table S4**. Somatic mutations and clonality analysis results
**Table S5**. TP53 immunohistochemistry cohort by TP53 status (referred to in Supplementary results)
**Table S6**. TP53 immunohistochemistry cohort by recurrence status (referred to in Supplementary results)
**Table S7**. General linear model for *TP53* mutation
**Table S8**. Imputed model for *TP53* mutation
**Table S9**. Multivariable Cox regression results for p53 IHC

## Data Availability

The sequencing dataset supporting the conclusions of this article is available under a data access agreement through the European Genome‐Phenome Archive with accession ID EGAS50000001298 (https://ega-archive.org/studies/EGAS50000001298).
