# Supplementary material for: Phylogenetic analysis of paired breast carcinomas identifies genetic events associated with clonal recurrence and invasive progression
Source: J Pathol. 2025 Oct 30;268(1):1–12. doi: 10.1002/path.6461 (PMC12699244; doi:10.1002/path.6461)
Supplement: Supplementary file 1 — Supplementary materials and methods Figure S1. Log ratio profiles generated by the Clonality package (R version 3.6.2) comparing a paired tumour based on total SCNA Figure S2. Discrepancy between the Clonality package and manual inspection for a non‐clonal case Figure S3. Example of three non‐clonal pairs Figure S4. Refphase profile of LP005 Figure S5. Correlation of various clinico‐pathological and genetic features with recurrence Figure S6. Correlation of time to recurrence and radiotherapy with recurrence Figure S7A. Illustration of the reasoning behind choosing phylogenetic analysis (with higher‐depth WES) over manual inspection of somatic copy number alterations (SCNAs) and breakpoints Figure S7B. Example of a clonal pair and a non‐clonal pair sequenced by whole exome sequencing (WES) Figure S8. Refphase profile for DCIS0276 WES (left) and WGS (right) Figure S9. Refphase profile for DCIS0506 WES (left) and WGS (right) Figure S10. Comparisons between primary and recurrent tumours Figure S11. Differences by recurrence histology Figure S12. Genomic features by recurrence Figure S13. Examples of clonal pairs with a truncal WGD event and a subclonal WGD event Figure S14. CNA frequency for selected chromosomes Figure S15. CNA frequency for chromosome 8 highlighting RAD21 (significantly different CN gain frequency between non‐recurrent and non‐clonal recurrence) and MYC (not different) Figure S16. Association of mutations with recurrence Figure S17. TP53 immunohistochemistry Figure S18. TP53 staining association with recurrence with all cases (left) and with cases also present in the genetic cohort (n = 10) removed (right) Table S1. Sample information Table S2. List of genes on targeted sequencing panel Table S3. Differences by recurrence clonality status Table S4. Somatic mutations and clonality analysis results Table S5. TP53 immunohistochemistry cohort by TP53 status (referred to in Supplementary results) Table S6. TP53 immunohistochemistry cohort by recurrence sta [file PATH-268-1-s001.zip › path6461-sup-0001-SuppMatMethFiguresS1-S18TablesS1-S9/path6461-sup-0001-SuppMatMethFiguresS1-S18TablesS2S3S5-S9.docx]

**Phylogenetic analysis of paired breast carcinomas identifies genetic events associated with clonal recurrence and invasive progression**

T Kader, M Zethoven, S Mahale *et al. J Pathol* <https://doi.org/10.1002/path.6461>

**Supplementary materials and methods**

**Supplementary results**

**Supplementary Figures S1–S18**

**Supplementary Tables S2, S3, and S5–S9**

**Supplementary Tables S1 and S4** **(provided as separate Excel files)**

Reference numbers refer to the main text list

**Supplementary methods and methods**

**Additional case description**

All but two of the recurrent tumours had the same ER status as the primary DCIS when data were available for both of the pair (supplementary material, Table S1). These two ER-positive primary DCIS (LP004, DCIS00498) recurred as ER-negative disease. Keeping in mind that IBC grade is not directly comparable to DCIS grade, the grade of recurrent tumours was not always similar to the primary DCIS. When grades of both were available (75/78 pairs), 23 out of 75 pairs (31%) recurred as a lower grade than the primary (i.e. high- or intermediate-grade DCIS recurred as low-grade DCIS or grade 1 IBC; e.g. DCIS00009: high-grade DCIS recurred as low-grade DCIS). On the other hand, 13 out of 75 pairs (18%) recurred as a higher grade than the primary (e.g. LP003: low-grade DCIS recurred as grade 2 IBC).

Cases 1783 and 2594 were treated by mastectomy and then recurred. This would be unusual in a contemporary cohort; however, these cases were diagnosed pre-2000. They were initially treated with breast-conserving therapy, but positive margins led to a second surgery and mastectomy.

**DNA extraction and sequencing**

Archival formalin-fixed, paraffin-embedded (FFPE) blocks of non-recurrent DCIS, all primary DCIS, and recurrent tumours were obtained from the associated hospitals. Haematoxylin and eosin-stained slides were reviewed by pathologist(s) to choose the best areas for microdissection. Stromal regions from ten breast samples were microdissected and used in each sequencing run as a pooled normal control and for validating rare variants. A summary of the cohorts, sequencing technologies, and aims is shown in Figure 1. Only DNA was used in this study and the Quant-iT^TM^ dsDNA High-Sensitivity Assay Kit (Invitrogen, Carlsbad, CA, USA) was used to determine the DNA concentration. The quality of DNA was assessed by a multiplex PCR assay with primer sets that produce 100–700 bp fragments from the *GAPDH* gene as previously described [9].

**Gene list of targeted sequencing panel (supplementary material, Table S2)**

*1. Mutated genes*. On the basis of the close genetic relationship between DCIS and invasive breast carcinoma (IBC) [33], it is likely that genes affected in IBC are also relevant for DCIS, and their presence in DCIS may indicate a subtype more likely to progress. Therefore, we evaluated 21 significantly mutated breast cancer genes with a frequency of mutation ≥ 2% based on analysis of The Cancer Genome Atlas (TCGA) data [37] and five additional genes based on the literature (e.g. *ESR1*).

*2. Invasive breast cancer copy number* (*CN*) *regions*. Fifty regions of CN change were included, comprising significant peaks identified by the TCGA breast cancer analysis (*q* < 0.001) and with frequency > 5% in the METABRIC study [37,38]. Most regions were represented by single genes, but some larger regions have multiple genes selected.

*3. DCIS CN regions*. Forty significant regions of CN change or allelic imbalance from our analysis of DCIS with and without recurrence were included [4]: regions significant at *p* < 0.002 in the all-types analysis, and *p* < 0.02 in the subgroup analyses, and also requiring significance in the TCGA analysis or >5% CN change frequency in the METABRIC study [38].

There was some overlap between these groups, enabling streamlining of design (107 genes in total).

**Data analysis**

Paired-end sequence reads from the targeted sequencing panel and whole exome sequencing (WES) were aligned to the g1k v37 (hg19) reference genome using BWA v0.7.17 [11]. Optical duplicate reads were removed using Picard (v1.119), and then local realignment around indels and base quality score recalibration were performed using the Genome Analysis Tool Kit (GATK version 3.8) [39]. SNP and indel variants were called using GATK Unified Genotyper, Platypus [40], and Varscan 2 [41]. Called variants were additionally annotated using the Ensembl Variant Effect Predictor (VEP), release 78 [12].

Somatic mutations in the tumour sequencing data were identified by applying the following filters: canonical transcript; variants identified by at least two variant callers, allele depth > 5 and read depth > 10 unless the variant was in *PIK3CA* or *TP53*. Since matched normal DNA was unavailable for this study for all samples, any variant with minor allele frequency present in the GnomAD non-cancer population (version 2.0) unless known to be pathogenic (e.g. *TP53*) was filtered out. Rare and uncommon pathogenic variants were validated along with their matched normal DNA, where available, by Sanger sequencing as previously described [6] to ensure their classification as a somatic mutation prior to clonality analysis. Any rare germline variant misclassified as somatic mutation could lead to a false clonality signal [42]. Therefore, this step of filtering out from the GnomAD non-cancer population was strictly maintained [20]. Variants reported in the literature as sequence artefacts were filtered out [43]. Manual inspection of the sequence reads using the Integrative Genomics Viewer [44] was performed before finalising the somatic mutations. Any false-positive variants due to sequencing artefacts were excluded from the final analysis.

Both off-target and on-target sequencing reads from the targeted panel were used to generate genome-wide copy number data using PureCN without matched normal [13]. However, 24 normal DNA samples were pooled and used as a baseline of PureCN for most WES cases (Exome v1) except for 12 primary–recurrent pairs for which only one normal DNA was used (Exome v2).

CNA profiles, purity, and ploidy status from solution 1 of PureCN data from WES were used to generate haplotype-specific copy number changes by multi-sample phasing using Refphase (<https://bitbucket.org/schwarzlab/refphase/src/master/>) as previously described [16]. Samples with insufficient depth and low quality failed to phase and were excluded (*n* = 2: cases DCIS00191 and DCIS00401). Haplotype-specific copy number changes were leveraged to infer phylogeny reconstruction and ancestral genomes between primary–recurrent pair by Minimum Event Distance for Intra-tumour Copy-number Comparisons-2 (MEDICC2 [17]) (*n* = 54/56).

For LCWGS, reads were aligned using BWA-MEM (v0.7.12-r1039) to hg19 (GRCh37) after removal of sequencing primers by cutadapt (v1.7.1) as previously described [8–10,15]. ControlFREEC (version 6.7) [14] was used to estimate copy number from the LCWGS data in 50 kb windows, with default parameters, no matched normal sample, and baseline ploidy set to 2. To reduce spurious calls, blacklisted regions as identified from Scheinin *et al* [43] were excluded. Fraction of genome altered (FGA) was calculated as previously described [4].

**Clonality analysis**

All sample log_2_ ratio raw data generated by PureCN were imported into Nexus (v10; BioDiscovery Inc., Hawthorne, CA, USA) and segmented using SNP-FASST with a stringency of 1 × 10^−8^ for WES and the targeted sequencing panel and 1 × 10^−5^ for LCWGS samples. Copy number gains were called if the log_2_ ratio of the segment was greater than 0.18 and losses were called if it was less than −0.18 as previously described [4,8,10].

**Clonality package**

A statistically based clonality analysis, Clonality, an R package (version 3.6), was used based on total CNAs and mutations [18,19]. This statistical approach was only used for cases run by the targeted sequencing panel for CNAs. All samples were run together in the package to generate the reference distribution [19]. The paired cases were analysed only when both primary and recurrent pairs were sequenced with the same technology. Since the package was developed based on low-resolution comparative genomic hybridisation and considers just one prominent CNA per chromosome arm, targeted sequencing panel data were reduced to ~10,000 locations as suggested. A *p* value of 0.01–0.05 was considered as equivocal; *p* < 0.01 was clonal; and *p* > 0.05 was non-clonal (supplementary material, Figure S1). Paired genome-wide copy number plots for all cases were generated as described in the package, and they all were manually verified using Nexus Copy Number^TM^ (v10; BioDiscovery Inc., Hawthorne, CA, USA). This manual verification was necessary for all cases. FFPE samples are notorious for spurious calls; therefore, any apparent rare or uncommon very small segment of gain/deletion might generate a low *p* value and be considered clonal by the package (supplementary material, Figure S2). Manual verification was also needed to rule out any sequencing-specific artefacts, such as 19p which often is seen as gained on the targeted sequencing panel (supplementary material, Figure S3). For somatic mutation, both the targeted sequencing panel and the WES data were considered. A *p* value generated by the Clonality package ‘*get.mutation.frequencies*’ function suggests a clonal pair if *p* < 0.05, with the TCGA breast cancer cohort used as a reference cohort as suggested by Mauguen *et al* [18]. The *p*-values generated by this package for both CNA and mutational profiles are recorded in the supplementary material, Table S4.

**Clonality index only for somatic mutations**

An additional estimation for clonal relatedness was performed as described by Schultheis *et al* [20], called clonality index (CI) and CI2. This approach was carried out for all cases run by the targeted sequencing panel and WES. The CI for a tumour pair was defined as

$\mathrm{CI} = \left\{ \begin{aligned} 1-\prod_{k = 1}^{n} f_{k}, n > 0 \\ 0, n = 0 \end{aligned} \right.$

where *f_k_* is the percentage of tumours in TCGA [37] harbouring a given mutation (*k*) and *n* is the number of shared mutations between a pair of tumours. The pair is considered genetically related if CI is more than 0.8.

CI2 is similar to CI, but by using an R package ROCR version 1.0-11 [45]. Unlike CI, where the threshold was fixed as 0.8 to define any tumour pair as ‘clonal’, the CI2 threshold will be carried out by the ROCR package. The threshold will vary based on the average mutation rate of that tumour type in the control dataset (TCGA-BRCA) as well as the frequency distribution of variants of the testing cohort. In our cohort, the generated threshold by the R package was 2.83, indicating that any pair with CI2 value > 2.83 was considered as clonal. Both CI and CI2 values are detailed in the supplementary material, Table S4.

**Phylogeny reconstruction using MEDICC2**

An evolutionary method was used for most paired cases in this study with high-depth WES data (*n* = 54/56) and WGS data (*n* = 4). Cases with less than 20× depth or low quality were excluded (*n* = 2: cases DCIS00191 and DCIS00401). Copy number profiles were pre-phased using Refphase to derive haplotype-specific CNA profiles [16] and MSAI, which was then used to derive phylogenetic trees for tumour pairs using MEDICC2 [17]. An example of a Refphase profile is shown in the supplementary material, Figure S4. All Refphase and MEDICC2 profiles were manually inspected to reduce overcalling clonality due to the presence of small shared segments, which were likely FFPE-related artefacts. We looked for clear evidence of CN breakpoints in both samples, and preferably evidence of allelic imbalance or LOH to support a CN event. In the absence of both features, shared MEDICC2 regions were called false. In addition, CN events in repetitive regions such as peri-centromeric heterochromatin or telomeres were discounted if precisely overlapping with these regions.

**Statistical analyses**

A *p*-value < 0.05 was considered significant unless stated otherwise. We used the Wilcoxon rank-sum test to compare continuous variables, and for categorical variables the *χ*^2^ test unless expected values were less than 5, in which case Fisher’s exact test was performed. Summary tables and associated statistical test results were generated using the R package *gtsummary* (version 1.7.2) [46]. Both univariable and multivariable Cox proportional hazards models were performed to identify clinical and molecular characteristics associated with patients’ recurrence risk (*survival::coxph* version 3.5-7) [25]. All cases with data for each characteristic were used in univariable analyses (*n* = 186); however, only the subset with complete data due to missing values was used in a multivariable analysis (*n* = 134). The proportionality assumption was checked using *cox.zph*. Internal model calibration and validity were performed using the bootstrap sampling approach in the *rms* package (version 6.7-1) [47]. Adjusted and unadjusted survival curves were generated using the survminer package version 0.4.9 (*ggadjustedcurves* and *ggsurvplot*) [48].

Figures were produced using BioRender (Toronto, Canada) or RStudio (version 2023.09.1; Posit Software, Boston, MA, USA) and assembled in Adobe Illustrator 2025. Copy number frequency plots and the OncoPrint image were created using *GenVisR* version 1.36 [36].

**Supplementary results**

One of our goals was to evaluate whether there were any differences between non-clonal and clonal primaries that might be able to predict the likelihood of a clonal *versus* non-clonal recurrence. However, the number of non-clonal primaries analysed by WES was too low for any such analysis. Our lower-coverage sequencing methods resulted in a higher proportion of non-clonal cases (more detail on this below), and including these would give us slightly more power to conduct this analysis. Therefore, we present the results of the comparison here including all cases, but with the caveat that it is possible that some of the recurrences are not truly non-clonal, but that we have lacked the resolution to determine their clonality status.

**Evidence for *de novo* development of ipsilateral recurrent tumours by low-resolution methods**

We investigated 26 tumour pairs (four contralateral) using a targeted sequencing panel (*n* = 20) or LCWGS (*n* = 1) or a combination (*n* = 5) due to limited DNA availability to test their clonal relatedness (examples in supplementary material, Figure S1). Therefore, while CNA profiles were available for all pairs, mutation analysis was only available for 20 pairs. Clonal relatedness was assessed for this cohort using the Clonality package, manual inspection of breakpoints, and clonality index (CI, CI2) (when mutation data were available).

Using copy number data, all contralateral tumours were genetically independent (i.e. non-clonal), as expected, while 68% (15/22) of ipsilateral primary–recurrent pairs were genetically related (i.e. clonal, representing true recurrences) and the remaining seven cases appeared to be independent tumours (i.e. non-clonal). Six of these seven cases had paired mutation data and all six pairs remained non-clonal when including these data. Out of these six cases, four pairs had no shared mutations or CNAs, and all of these carried private CNAs or mutations. Two out of these four pairs had private mutations in both primary and recurrent tumours, further evidence for the second tumours to be new primaries and verifying that the degree of normal cell contamination was not masking shared events. One pair (case 3933) had two shared copy number events but with different breakpoints, including 16q loss, one of the most common copy number events of IBC (Clonality package *p* = 0.98) (supplementary material, Figure S3). The remaining pair (case 3936) had no shared CNAs but shared a *PIK3CA* (H1047R) mutation, the most common mutation of IBC. This result suggested a non-clonal pair by Clonality package and clonality index (*p* = 0.074, CI = 0.8, CI2 = 2.83) (supplementary material, Table S4).

Two cases analysed by WES were unable to achieve a high sequencing depth (DCIS00191, DCIS00401). Clonality analysis was performed for these two cases by clonality index and manual inspection of copy number (supplementary material, Table S4), which suggested clonal cases. Overall, 7/24 cases by lower-resolution methods were non-clonal (29%). When combined with the high-resolution WES/WGS cases, the total number of non-clonal cases was 11/78 (14%).

**Association of clonality status with clinico-pathological features**

The clonality status (clonal recurrence *versus* non-clonal recurrence) was not significantly associated with the type of recurrence (IBC/DCIS), or the grade, ER status or HER2 status of the primary DCIS (supplementary material, Table S2 and Figure S5). The age at diagnosis did not affect the type of recurrence (clonal median 60.0, non-clonal 59, *p* = 0.8). Only the size of the primary DCIS was significantly different between non-clonal (median 7.6 mm, range 2–17 mm) and clonal DCIS (median 15.5 mm, range 1.8–80 mm, *p* = 0.023), but was not statistically significantly different to non-recurrent DCIS (median 15 mm, range 2–48 mm) (supplementary material, Figure S5). Previously, in a study of synchronous DCIS/IBC, we identified 3/21 cases that were non-clonal. All three non-clonal DCIS were in a different block to the IBC and were contained within tumours greater than 25 mm in size [27]. Therefore, non-clonality is unlikely to be due to the presence of polyclonal DCIS at diagnosis. In addition, there was no significant difference in TILs among clonal primary (median 10%, range 0–90%), non-clonal primary (median 5%, range 3–5%), and non-recurrent (median 5%, range 1–30%) (*p* = 0.32, Kruskal–Wallis rank-sum test) (supplementary material, Figure S5).

One hypothesis that we had was that later recurrences would be more likely to be independent tumours. However, the median time to detecting the second tumour was not significantly different: 3.3 years for clonal and 4.7 years for non-clonal recurrences, respectively (*p*= 0.6, Wilcoxon rank-sum test; supplementary material, Figure S6). Indeed, there were eight unequivocally clonal recurrences occurring more than 10 years after the initial diagnosis, five IBC and three DCIS. All shared at least two cancer-driving mutations and/or copy number breakpoints and also had divergent events such as additional variants or copy number changes.

Another hypothesis that we had, that breast tumours recurring after radiotherapy (RT) would be more likely to be non-clonal due to the DNA-damaging effects of RT, was not supported by our data (*p*= 0.7), although our power was extremely limited (RT+/clonal 12/62; RT+/non-clonal 1/10) [49]. However, the only difference in clinical or histological features that indicated the likelihood of a non-clonal recurrence compared with no recurrence was that non-clonal recurrences were less likely to have had treatment with radiotherapy (*p*= 0.014; supplementary material, Figure S6).

**Comparison of non-clonal primaries with non-recurrence cases**

Non-clonal cases had CNAs and mutational status similar to those of non-recurrent except for a few changes on chromosome 8 (excluding the oncogene *MYC*) (supplementary material, Figure S15). Power was limited due to the small number of non-clonal recurrence cases: non-clonal recurrences had a lower FGA and smaller size than did non-recurrences, but neither was statistically significant (supplementary material, Figure S12). Ploidy and whole genome duplication rate were not different. Non-clonal primary DCIS had many fewer CN events, with 6/11 cases having < 5% of the genome affected, compared with 5/67 clonal primaries and 8/32 non-recurrent DCIS. Four of the six non-clonal DCIS with low CNAs also had no somatic variants, which could potentially be due to increased normal cell contamination in some cases.

**Similarities between primary and recurrence depending on clonality**

When looking at the consistency of clinico-pathological features between paired primary and recurrence depending on clonality status, there was no significant difference in the TILs between primary and clonal/non-clonal recurrences (*p*> 0.05 for both, paired Student’s *t*-test; supplementary material, Figure S10). There was also no difference in whether grade was consistent – when considering just the low–high mismatches, only four were detected, all between clonal primaries and their recurrences. A stricter application of grade consistency (e.g. low to intermediate called a mismatch) found that 5/11 non-clonal primaries matched their recurrences, and 35/64 clonal matched theirs (*p*= 0.75, two-tailed Fisher’s exact test). ER status was also consistent regardless of clonality, with 9/9 non-clonal recurrences and 54/56 clonal recurrences showing the same ER status. Therefore, neither grade nor ER status is a good indicator of clonality.

In contrast to the clonal recurrences, FGA of non-clonal recurrent cases showed no significant difference between the matched pairs (median FGA 5% *versus* 10.6%, respectively) (*p*= 0.89, paired Student’s *t*-test) (supplementary material, Figure S10). Ploidy was slightly lower in non-clonal recurrences when compared with their primaries, but this was not statistically significant (*p*= 0.095, paired Student’s *t*-test).

**Association of *TP53* with recurrence**

1. *Models with genetic data*

We tested *TP53* mutation alone (supplementary material, Figure S16) in a multivariable logistic regression model with ER status, grade, and radiotherapy. Results from the multivariable logistic regression model showed that the OR for *TP53* mutation increases, but with very wide confidence intervals (supplementary material, Table S7). ER status has limited effect on the model. It is likely that including these additional factors has led to overfitting, particularly given that there is substantial missing data for radiotherapy treatment (20/99 cases missing this variable). Therefore, we also evaluated a multiple imputed model. ER added little to the model and could be removed (supplementary material, Table S8).

1. *Immunohistochemistry*

To validate the prognostic value of *TP53* mutations, we performed TP53 IHC. We first evaluated the accuracy of assessing *TP53* mutation status by IHC in DCIS. There were 35 cases available with both mutation and protein data, including 11 samples with a *TP53* mutation and 24 samples without. Protein staining was concordant with the mutation status in 31/35 (89%) cases (supplementary material, Figure S12). Of the three mutated cases called as normal, two could be explained by sample heterogeneity as both had mutation allele frequencies of < 0.2%. The remaining case had a truncating mutation with an allele frequency of 0.71 (p.Lys132Ter) that would have been expected to lead to protein absence. One case lacking a mutation was called as complete absence staining, which could be due to fixation issues, as only a few non-tumour cells stained faintly. Overall, the concordance was sufficiently strong to proceed to a larger-scale analysis using cases treated with breast-conserving surgery, with or without radiotherapy (RT). Abnormal TP53 staining was seen in 36/186 scorable cases (19.4%) and was associated with higher nuclear grade, negative ER and PR staining, and positive HER2 staining, but not with tumour size or patient age (supplementary material, Figure S17 and Tables S5 and S6).

Abnormal TP53 status was significantly associated with ipsilateral recurrence in a univariable analysis (*p* = 0.041, HR = 1.91, 95% CI 1.03–3.55; supplementary material, Figure S18). As expected, younger age was also associated with an increased risk of recurrence, while treatment with radiotherapy was associated with decreased recurrence (HR = 0.27; 95% CI 0.11–0.64). Grade was not significantly associated with recurrence. Because of the possibly confounding association between *TP53* mutation and high grade/ER status/HER2/PR status, we tested a multivariable model that included these factors, as well as age and radiotherapy (*n* = 134 cases with complete data; supplementary material, Table S9). TP53 status (HR = 3.13; 95% CI 1.26–7.8) and radiotherapy (HR = 0.37; 95% CI 0.14–0.99) were significantly associated with recurrence (supplementary material, Figure S18). Because of the overlap with the discovery cohort, we tested a model that excluded ten cases with genetic data (nine with recurrence and one non-recurrent). The multivariable model was no longer statistically significant with these cases removed (supplementary material, Figure S18 and Table S9).

We also tested a model that included an interaction between TP53 and radiotherapy, given the known association of TP53 with the DNA damage response and radiation [50] (supplementary material, Figure S11 and Table S5). This model had a better concordance and validity compared with the model without the interaction (*p* = 0.009, C-index 0.72 *versus* 0.69). When removing the overlapping cases, this interaction was not statistically significant (*p* = 0.051, C-index 0.74). A simplified model with only p53, grade, age, radiotherapy, and the interaction between radiotherapy and p53 was the most statistically significant in the full and independent case cohorts (both *p* < 0.001, concordance of 0.74 and 0.745, respectively; supplementary material, Table S9).

**Conclusions**

We were unable to identify any primary tumour or patient characteristics that could indicate non-clonal recurrence, although we lacked family history and other indicative epidemiological information that might inform risk. TP53 abnormality may be associated with risk of recurrence, particularly as related to radiotherapy, but needs to be verified given the limited cases and lack of case–control matching in our cohort.


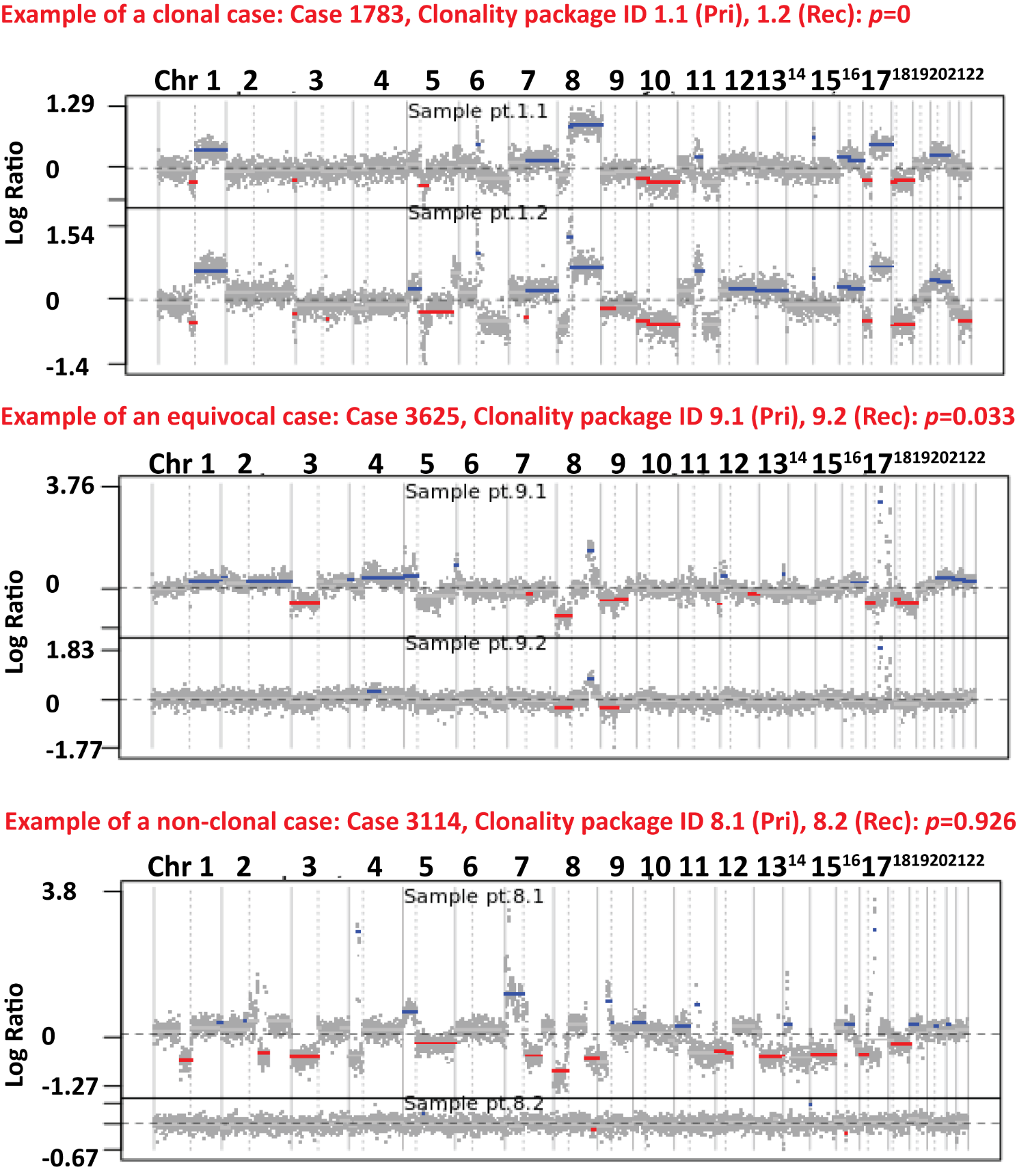


**Figure S1.** **Log ratio profiles generated by the Clonality package in R (version 3.6.2) comparing a paired tumour based on total SCNA.** Here, we show an example of a clonal (*p* < 0.01), an equivocal (*p* = 0.01–0.05), and a non-clonal case (*p* > 0.05). Blue indicates a gain/high gain/amplification of chromosomal segments/arm and red indicates loss of chromosomal segments/arm. The data were generated by the targeted sequencing panel.


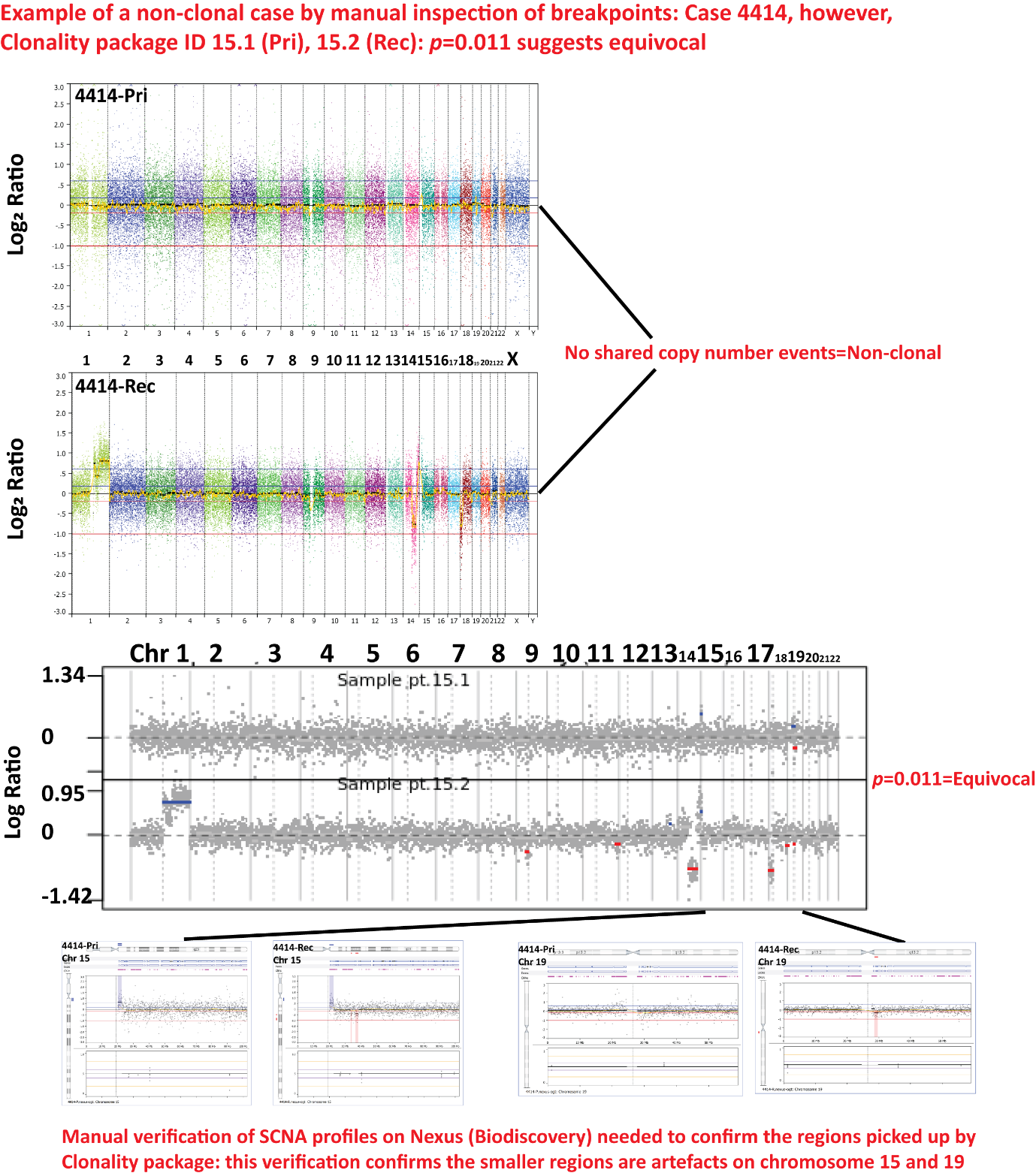


**Figure S2. Discrepancy between the Clonality package and manual inspection for a non-clonal case.** Here, we show an example of a non-clonal case by manual inspection of CNAs and breakpoints but defined as equivocal (*p* = 0.01–0.05) using the Clonality package. Manual verification of those independent segments for chromosomes 15 and 19 suggests either centromeric region or sequencing artefacts of FFPE. These segments were being used by the Clonality package and contributed to a clonality call. However, by rechecking these small segments manually, most likely they were not real changes.


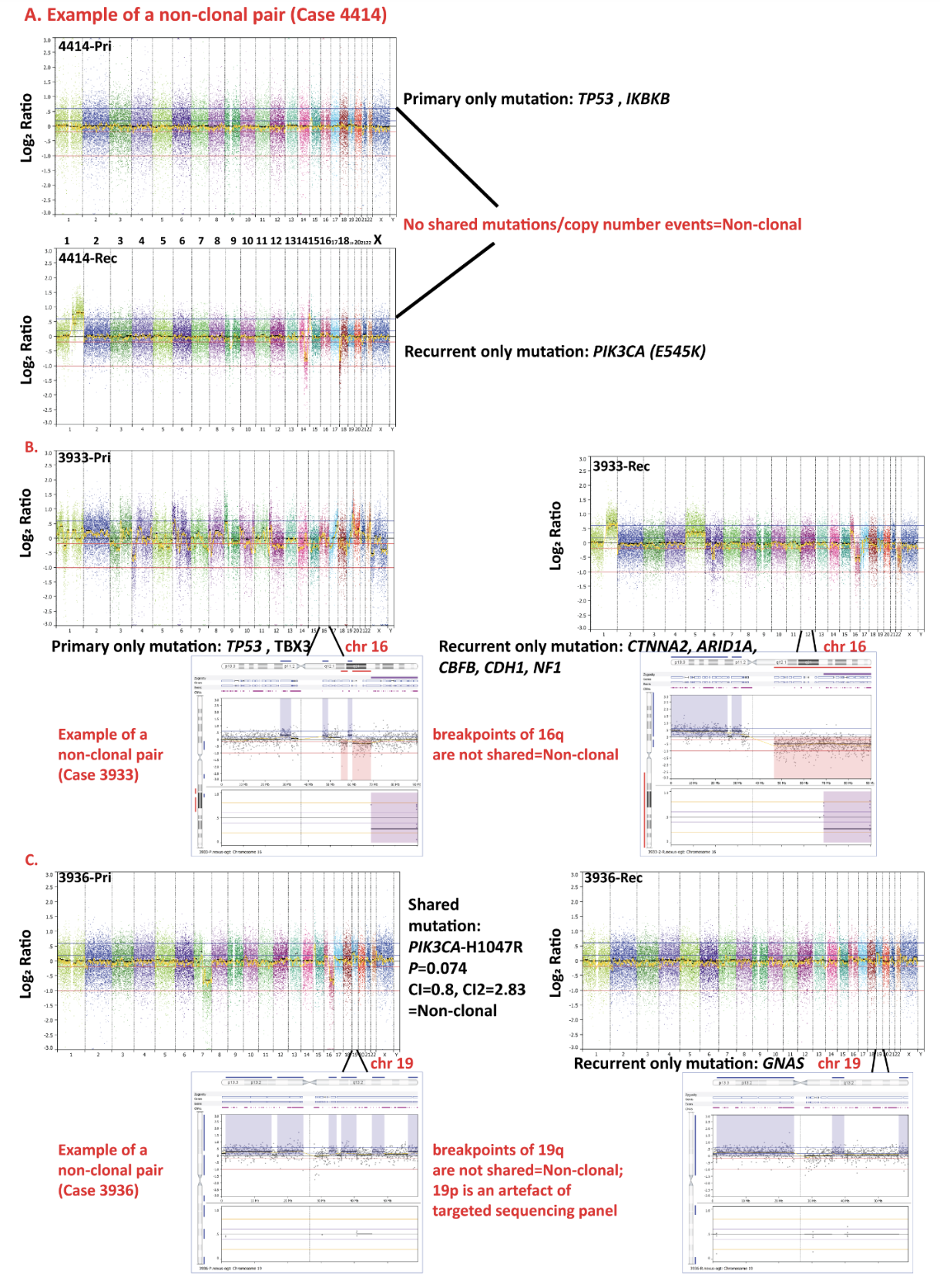


**Figure S3.** **Example of three non-clonal pairs.** (A) Case 4414, (B) case 3933, and (C) case 3936. The cases were classified as non-clonal, based on manual inspection of CNAs and breakpoints as well as using the Clonality package and indices. All modalities confirmed a lack of shared CNAs and/or somatic mutations: *p* > 0.05 by Clonality package; clonality indices CI and CI2 of ≤ 0.8 or ≤ 2.83, respectively. Here, we also show an example of technology-specific artefacts.


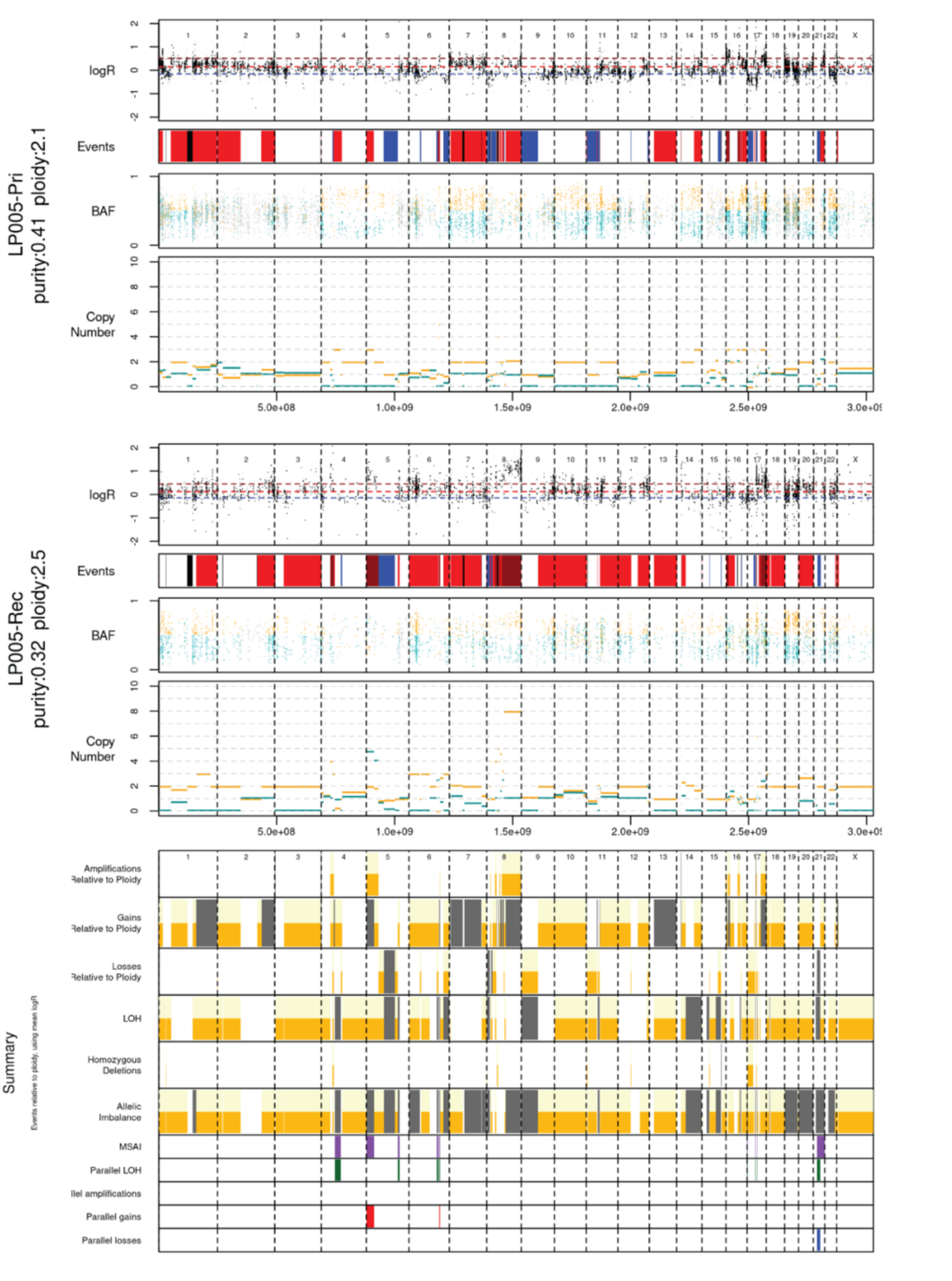


**Figure S4. Refphase profile of LP005.** Log ratio (logR) and B-allele frequency (BAF) profiles are shown as well as haplotypes using different colours on copy number profiles in yellow and green. The summary profiles include mirrored subclonal allele imbalance (MSAI). Parallel LOH, parallel gains, or losses are shown as well.


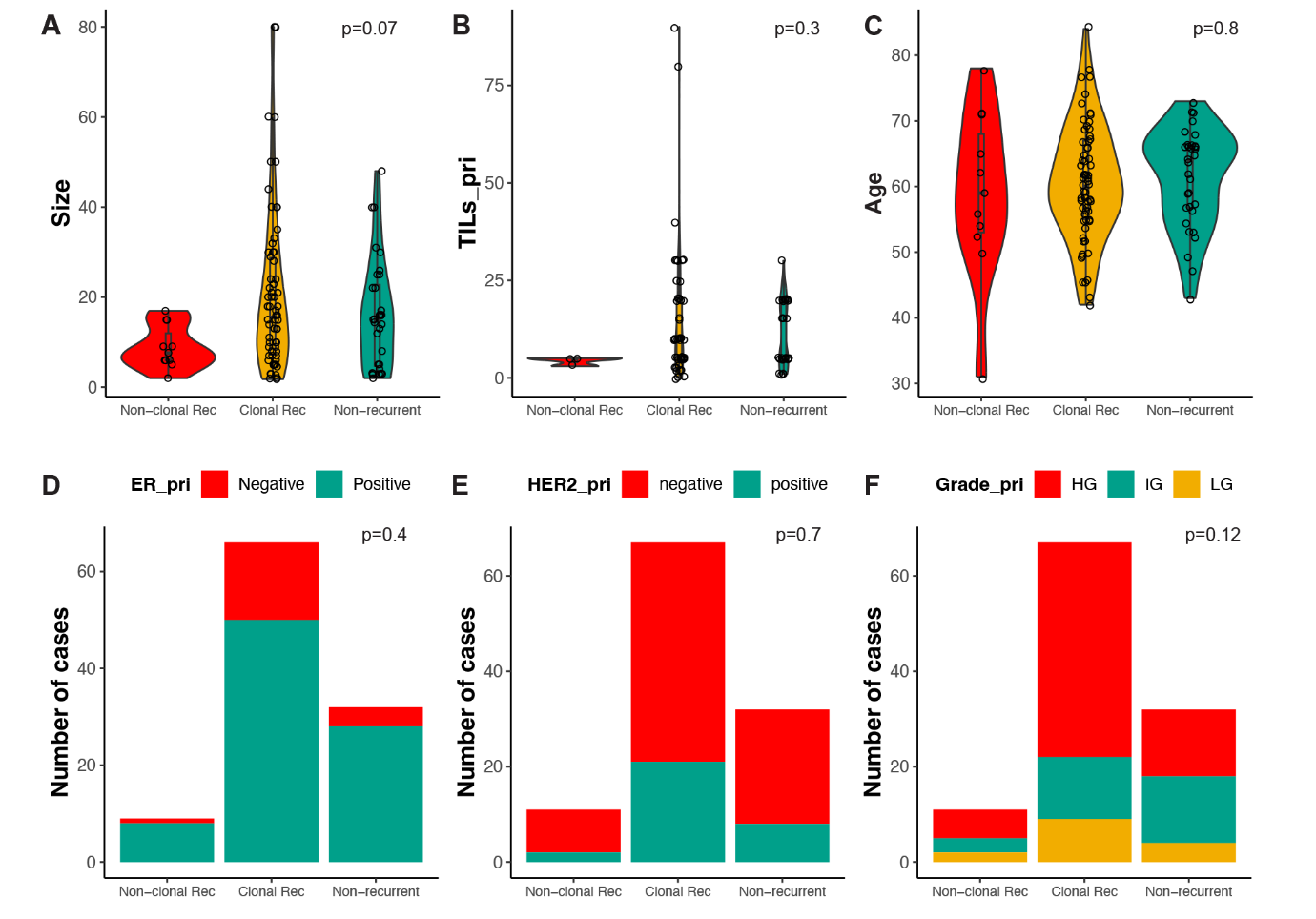


**Figure S5. Correlation of various clinico-pathological and genetic features with recurrence. (**A) Size in mm. (B) Tumour lymphocytes (%). (C) Age. (D,E) ER and HER2 status of primary DCIS. (F) Grade of primary DCIS. HG, high grade; IG, intermediate grade; LG, low grade.


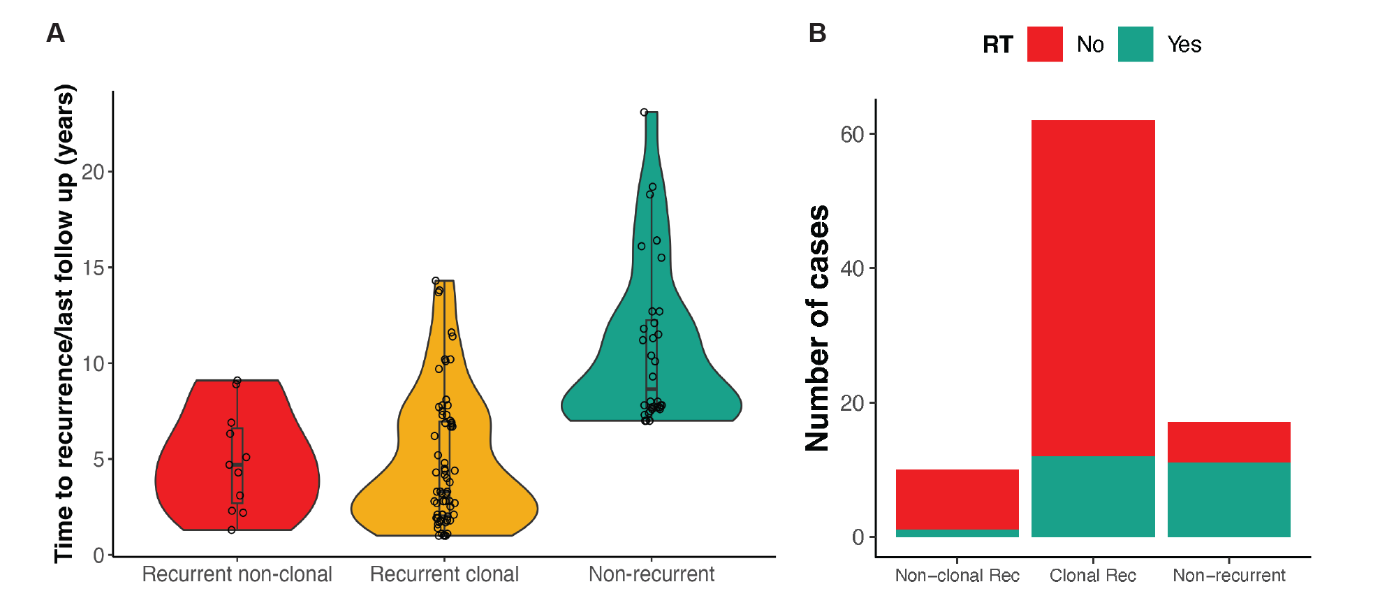


**Figure****S6. Correlation of time to recurrence and radiotherapy with recurrence (**A) Time to recurrence or last follow-up by clonality status. (B) Whether the primary DCIS was treated with adjuvant radiotherapy (RT).


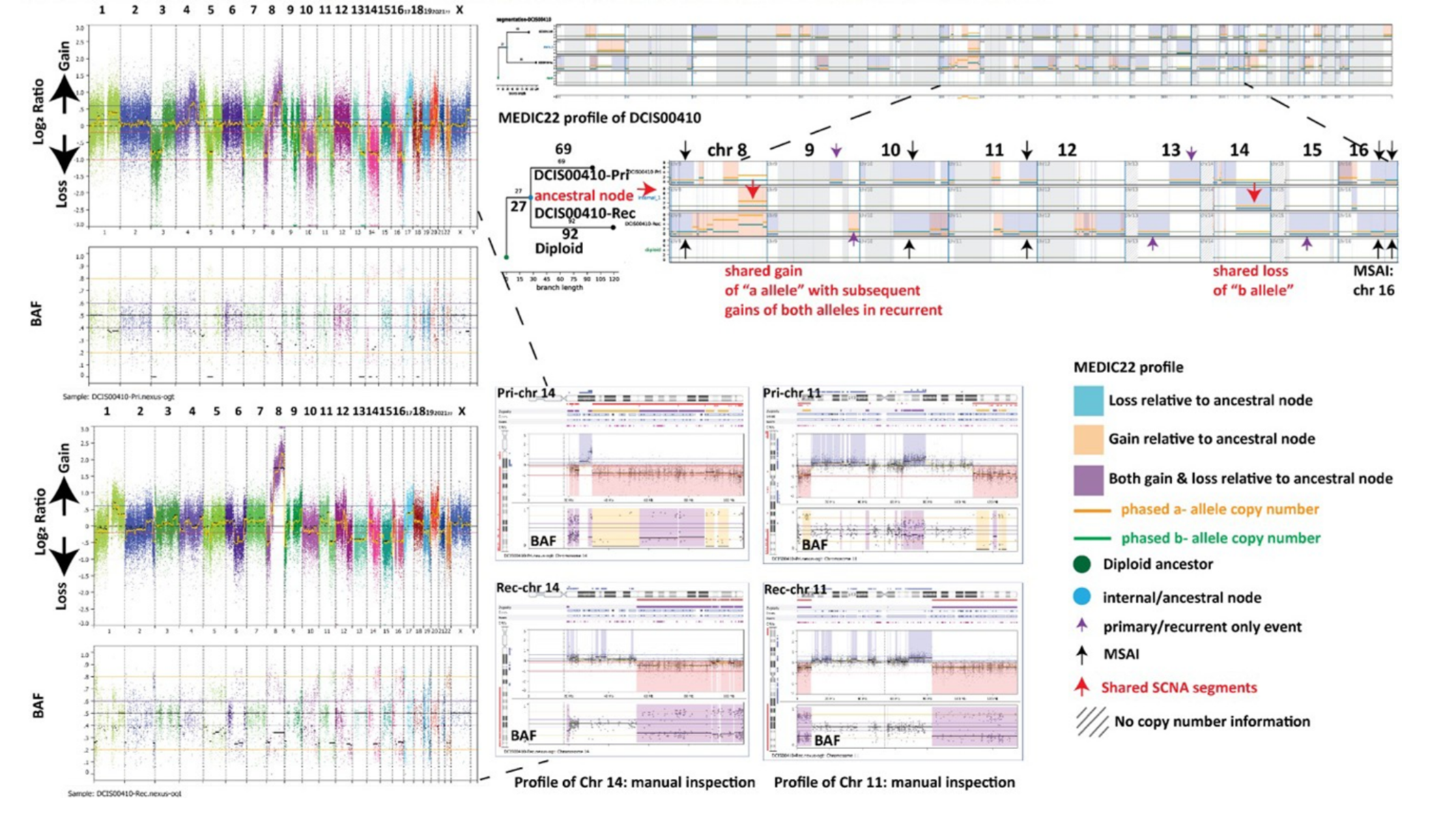


**Figure S7A.** **Illustration of the reasoning behind choosing phylogenetic analysis (with higher-depth WES) over manual inspection of somatic copy number alterations (SCNAs) and breakpoints.** The latter method could conclude a high number of cases as non-clonal. Investigating the evolutionary history of a tumour pair, on the other hand, revealed the presence of the ancestral genome with subsequent gains and losses and can distinguish clonal and subclonal events. As opposed to looking at any chromosomal event as a single event, phylogenetic analysis reveals the evolutionary history of SCNA segments. The log_2_ ratio profile of this case, DCIS00410, showed multiple chromosomal changes but different breakpoints, such as chromosomes 11, 14, and 8. Therefore, even with allele-specific SCNAs indicated by the B-allele frequency (BAF profile), this case was defined as non-clonal by manual inspection. Profiles of chromosomes 11 and 14 based on allele-specific SCNAs are shown here as well (top panel Log_2_ ratio profile: red = loss, blue = gain; bottom BAF panel: yellow = LOH, purple = AI), confirming that the breakpoints were different. In contrast, Refphase allowed us to generate haplotype-specific copy numbers and therefore clarified why we observed mismatched breakpoints on the same chromosome between pairs by manual inspection. When we looked at the MEDICC2 profile, chromosome 11 showed an MSAI (black arrow) (different allele was lost in this case), which explained the different breakpoints on manual inspection. When we looked at chromosome 14, it was clear that loss of a partial chromosome 14 (b-allele) was shared (a-allele = 1 copy; b-allele = 0 copy) between the pair (red arrow) with further b-allele loss for the rest of the chromosome 14 arm only in the primary DCIS, but not in the recurrent tumour (i.e. subclonal). In this figure, some of the other shared events, MSAI, and subclonal events are indicated by red, black, and purple arrows, respectively.


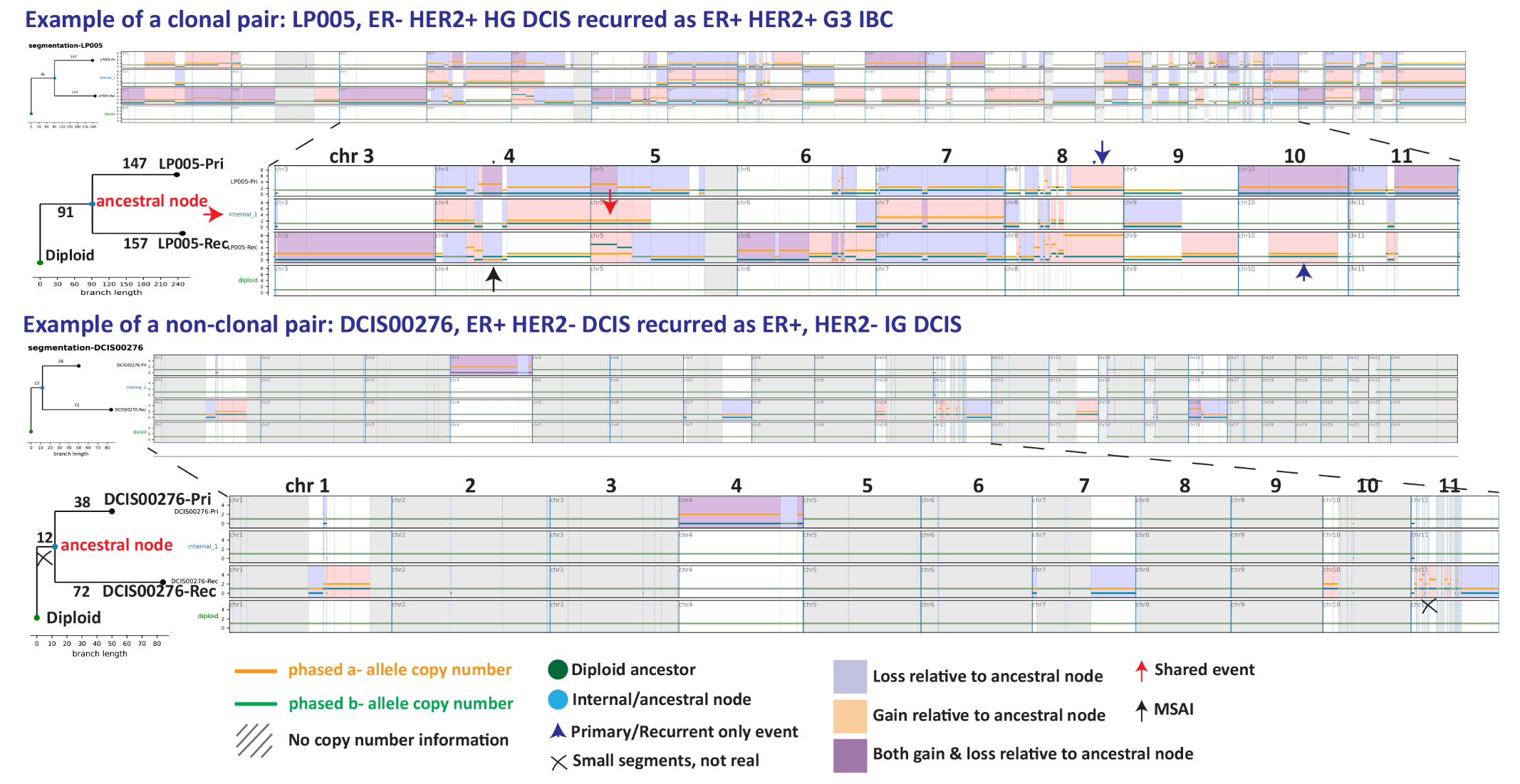


**Figure S7B. Example of a clonal pair and a non-clonal pair sequenced by whole exome sequencing (WES).** These profiles were generated by MEDICC2. Firstly, in case LP005, the phylogenetic tree suggests that there were 91 clonal segments identified in the ancestral genome (internal node), indicating truncal events in the primary DCIS and recurrent IBC. The recurrent tumour had 157 divergent CNA segments and the primary had 147, indicating events for both tumours that occurred after the truncal events (examples: purple arrows). Chromosomes 3–18 are magnified for visualisation. For example, gains of an ‘A-allele’ of the first three segments of chromosome 5 (‘A’ allele had 2 copies while ‘B’ allele had 1) were shared between the primary and recurrent. Then there was a further gain of the A-allele (from 2 copies to 3 copies) and loss of heterozygosity of the B-allele (1 copy to 0) for the first four segments in the primary DCIS. On the other hand, the first two segments of chromosome 5 in the recurrent IBC had gain of the B-allele to 4 copies and the first segment gained one more time for a total of 5 copies while the A-allele remained at 2. An example of mirrored subclonal allele imbalance (MSAI) is shown on chromosome 4 (black arrow), suggesting loss of different alleles on the same CNA segments (i.e. parallel evolution). For case DCIS00276, a few small segments were detected as truncal events (truncal segments = 12). We manually inspected their location in the copy number profile, which suggested that they are not real segments. Lack of shared CNAs and mutations suggested that this pair was non-clonal, which was confirmed by whole genome sequencing (supplementary material, Figure S8).

**
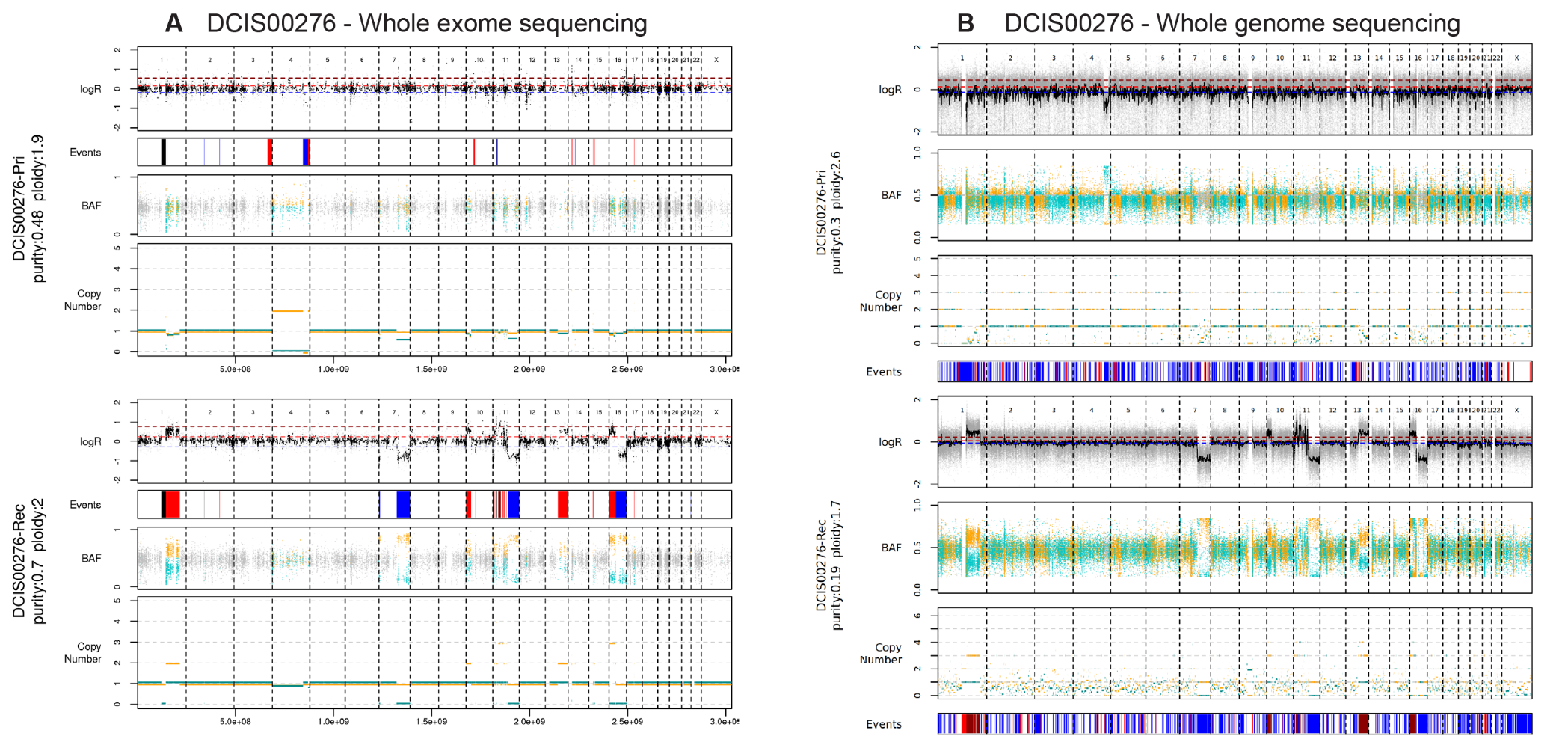
**

**Figure S8. Refphase profile for DCIS0276 WES (left) and WGS (right).** The non-clonal status of this case was confirmed with the higher-resolution data.

**
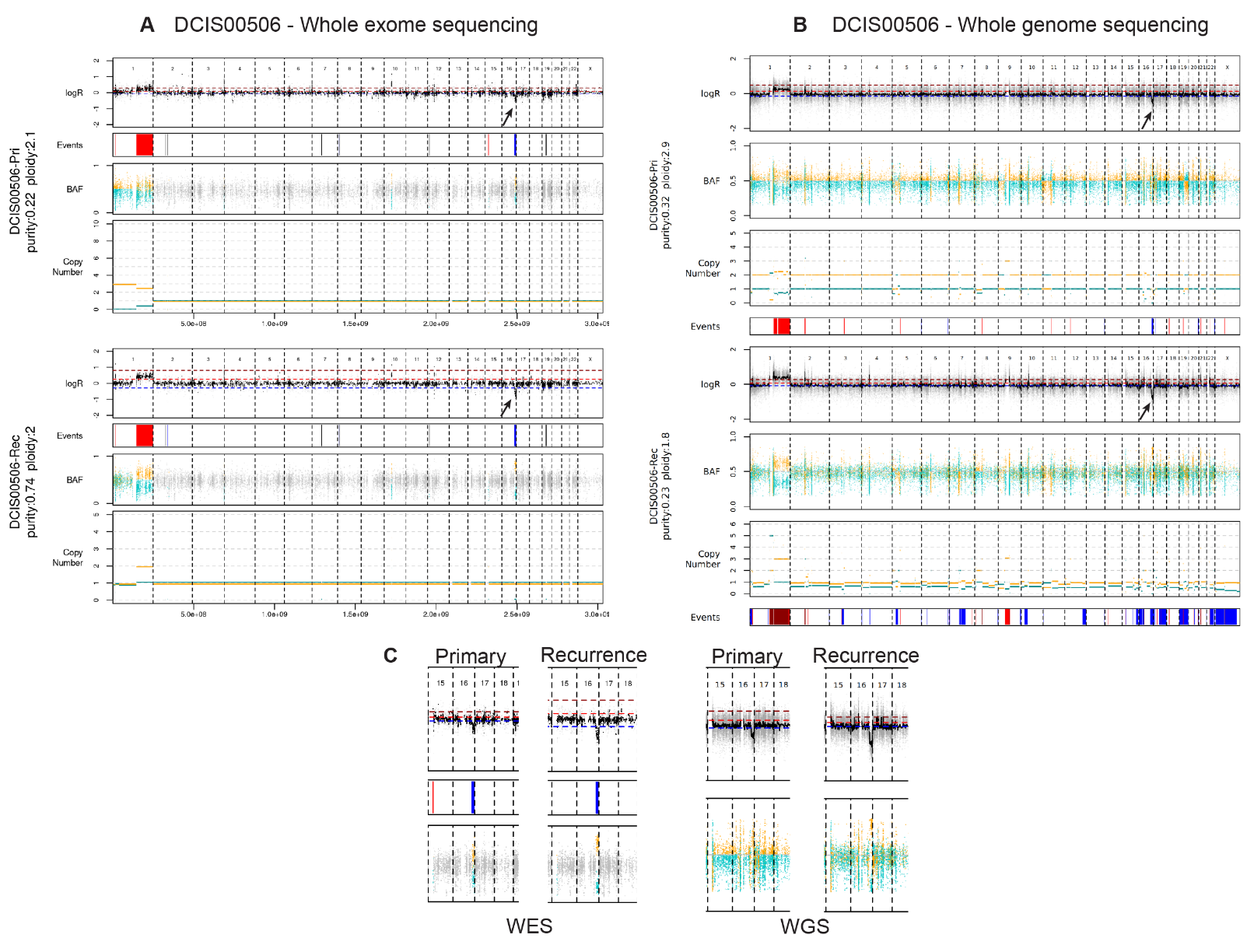
**

**Figure S9. Refphase profile for DCIS0506 WES (left) and WGS (right).** With increased resolution, a deletion at 16q (arrow and magnified below) was clarified as being genuine, and in concert with the shared 1q gain and *PIK3CA* mutation, this case was changed from non-clonal to clonal.


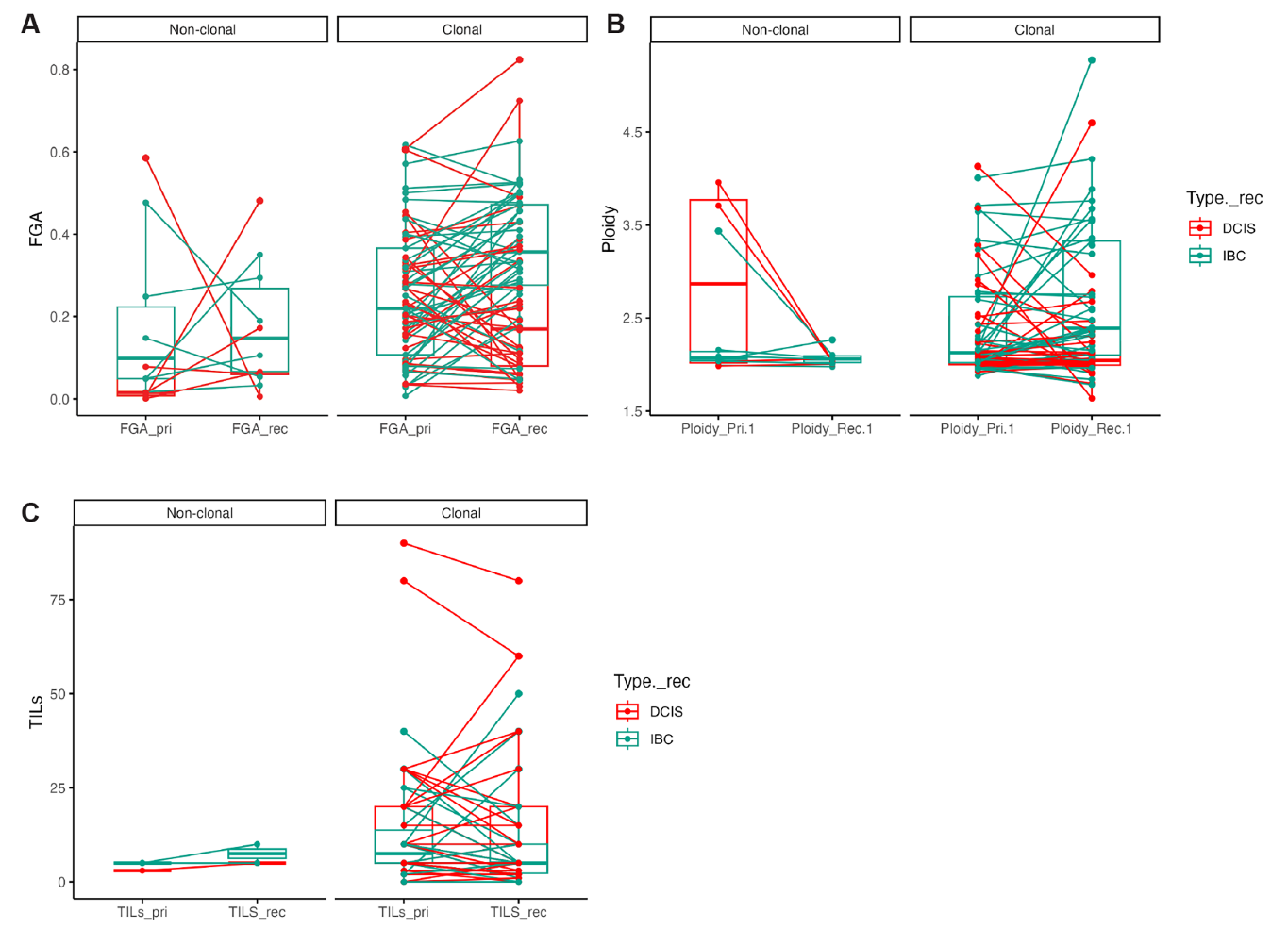


**Figure S10. Comparisons between primary and recurrent tumours.** (A) Fraction of the genome altered (FGA), comparing pairs of primary and recurrence tumours, separated by clonal status and coloured by recurrence type (DCIS, IBC). (B) Tumour genome ploidy, comparing pairs of primary and recurrence tumours, separated by clonal status and coloured by recurrence type. (C) Difference in tumour-infiltrating lymphocytes (TILs) separated by clonal status and coloured by recurrence type.


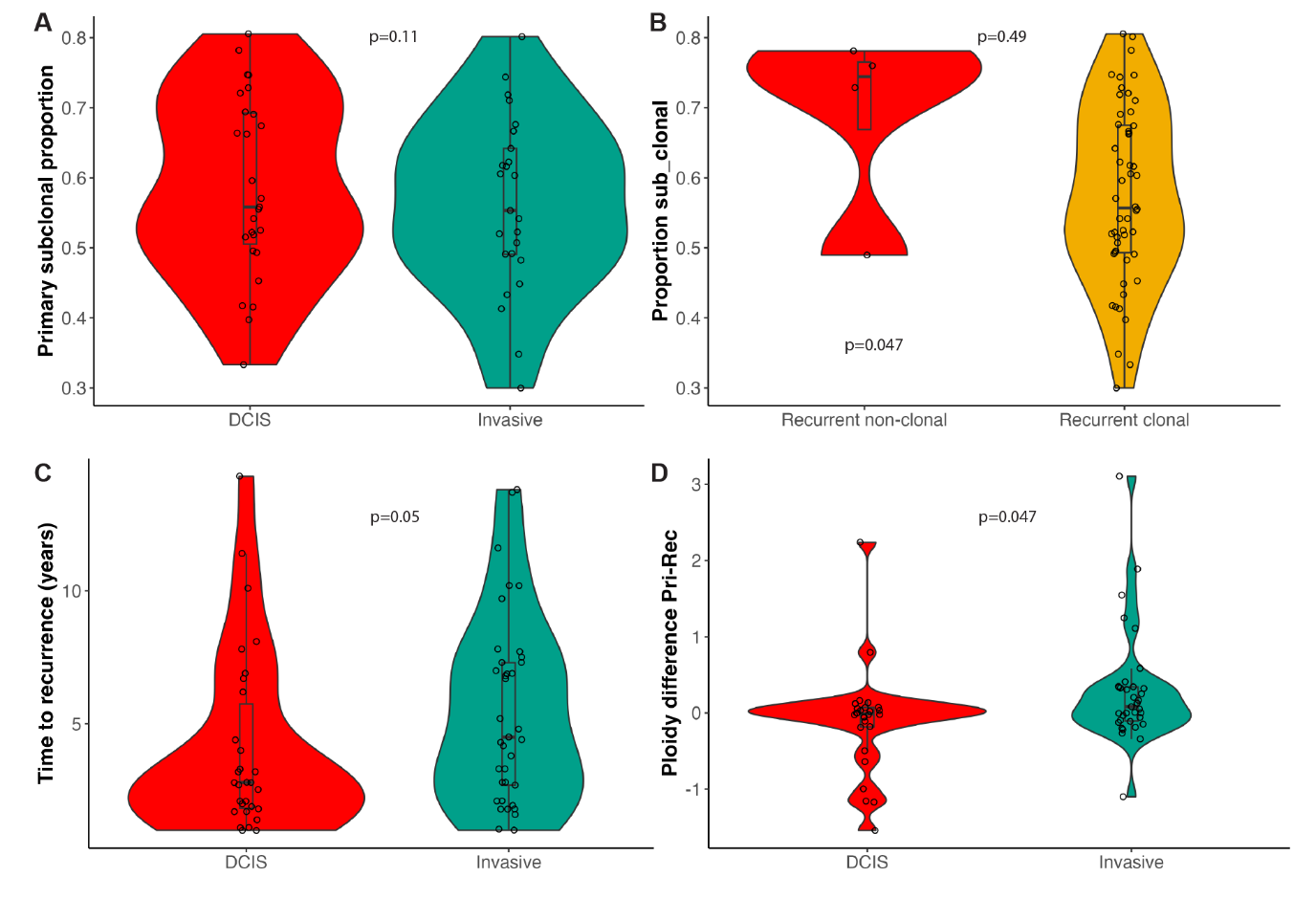


**Figure S11.** **Differences by recurrence histology**. (A) Proportion of the primary DCIS called as subclonal by MEDICC2. *p* value from Wilcoxon test. (B) Proportion of the primary DCIS with clonal recurrences called as subclonal by MEDICC2, separated by recurrence type. *p* value from Wilcoxon test. (C) Difference in time to clonal recurrence by recurrence type. *p* value from Wilcoxon test. (D) Difference in ploidy between primary DCIS and their clonal recurrences, comparing DCIS with IBC recurrences. *p* value from Wilcoxon test.

**
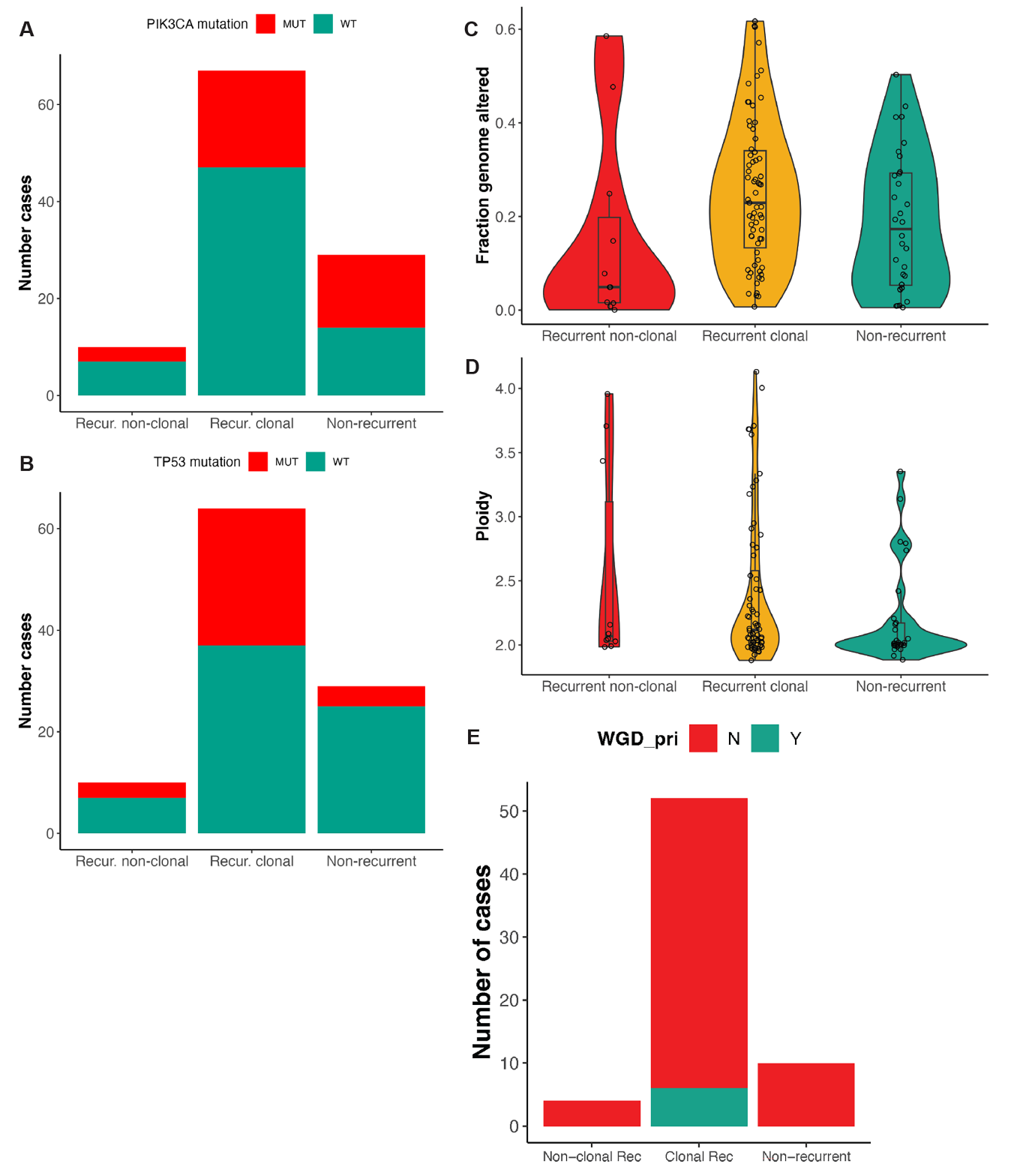
**

**Figure S12. Genomic features by recurrence.** (A) *PIK3CA* mutations. (B) *TP53* mutations. (C) Fraction genome altered (FGA). (D) Ploidy. (E) Whole genome duplication (WGD). MUT, mutated; WT, wild type (no mutation); N, no WGD present; Y, yes WGD present.


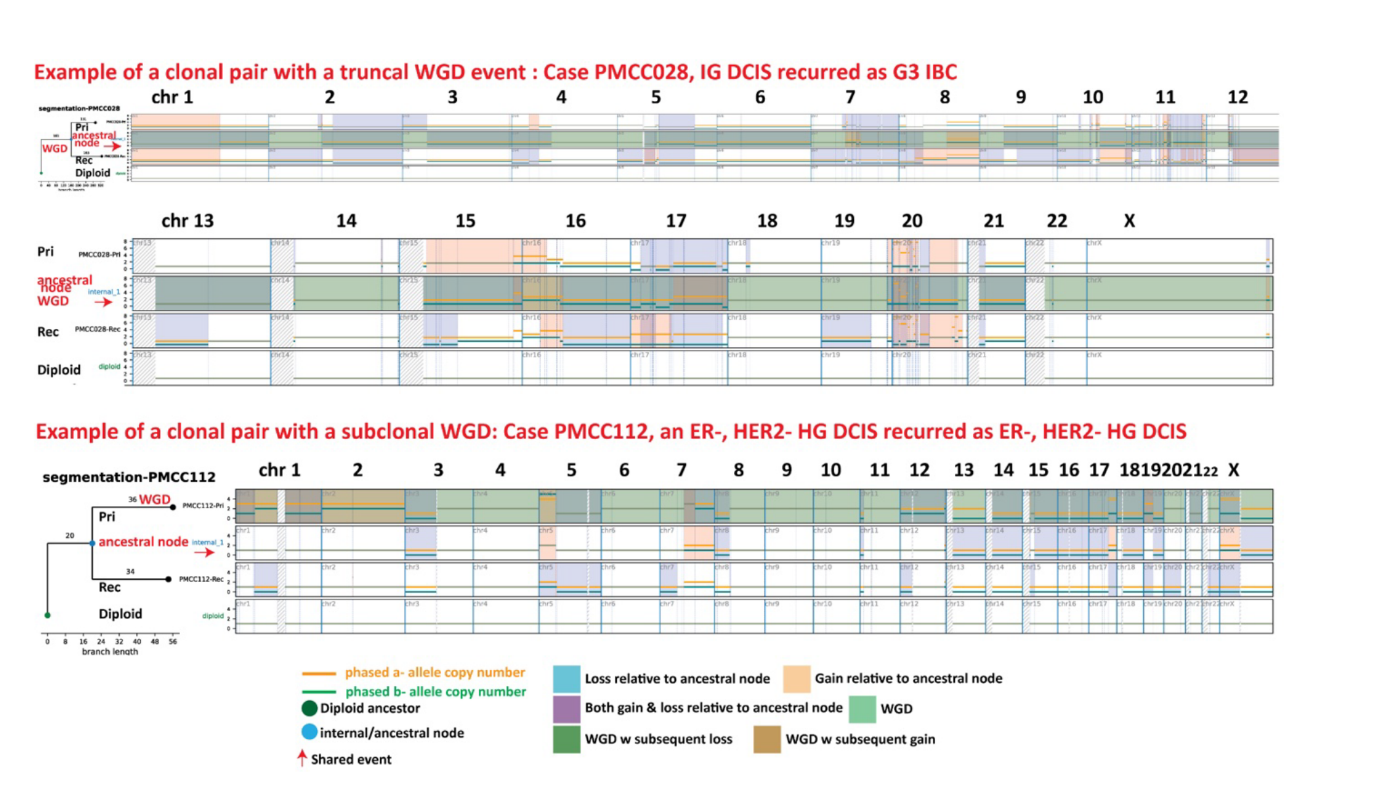


**Figure S13. Examples of clonal pairs with a truncal WGD event and a subclonal WGD event.** These profiles were generated by MEDICC2 based on haplotype-specific copy number profiles. Top: firstly, the phylogenetic tree of case PMCC028 suggested a WGD event in the ancestral genome (internal node), indicating a truncal WGD event between the primary DCIS and recurrent IBC. For example, chromosome 14 in the ancestral genome was shown as doubled (from 1 copy of each allele to 2 copies of each allele) (diploid = 1 copy of ‘a’ allele and 1 copy of ‘b’ allele representing 1 copy each from each parent). In addition, there were subsequent gains or losses in the ancestral genome. For example, there was a loss of b allele in chromosome 21 after a WGD event, indicated as b allele = 1 copy, while a allele remained as 2 copies. This profile also suggested that there were a large number of ancestral events (161) along with subclonal segments (131 or 165), suggesting an ongoing chromosomal instability. Bottom: PMCC112, on the other hand, showed a subclonal WGD event (primary only). For example, both alleles of chromosomes 4, 9, 10, and 11 have become 2 copies each from 1 copy each. Subsequent gains and losses of some chromosomes were also observed, for example chromosome 2.


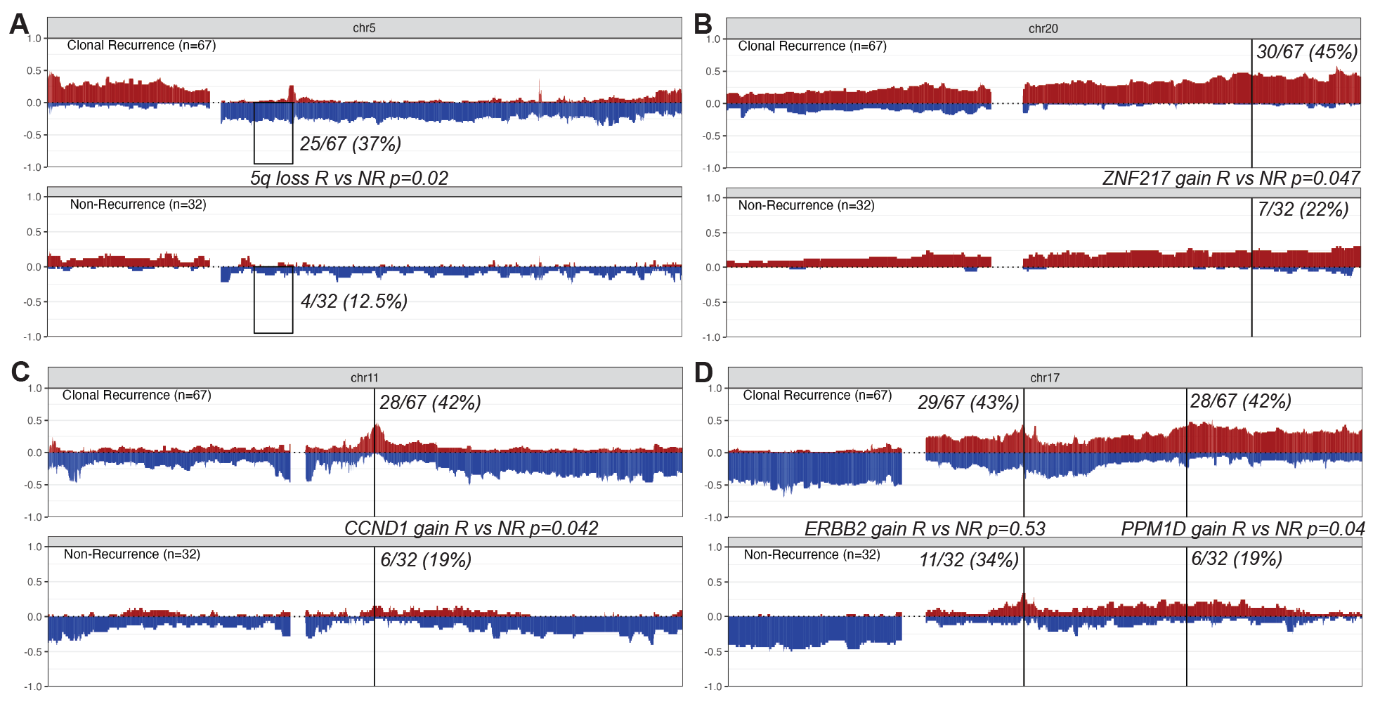


**Figure S14. CNA frequency for selected chromosomes**. Each plot shows the frequency of copy number gain (red) and loss (blue) in primary DCIS with a clonal recurrence and non-recurrence. Vertical lines indicate genes of interest. (A) Chromosome 5. Box indicates region of most significant difference in CN loss frequency between clonal recurrent DCIS and non-recurrent. (B) Chromosome 20 (*ZNF217* gain). *p* values from *χ*^2^ tests.(C) Chromosome 11 (*CCND1* gain). (D) Chromosome 17 (*ERBB2* and *PPM1D* gain).


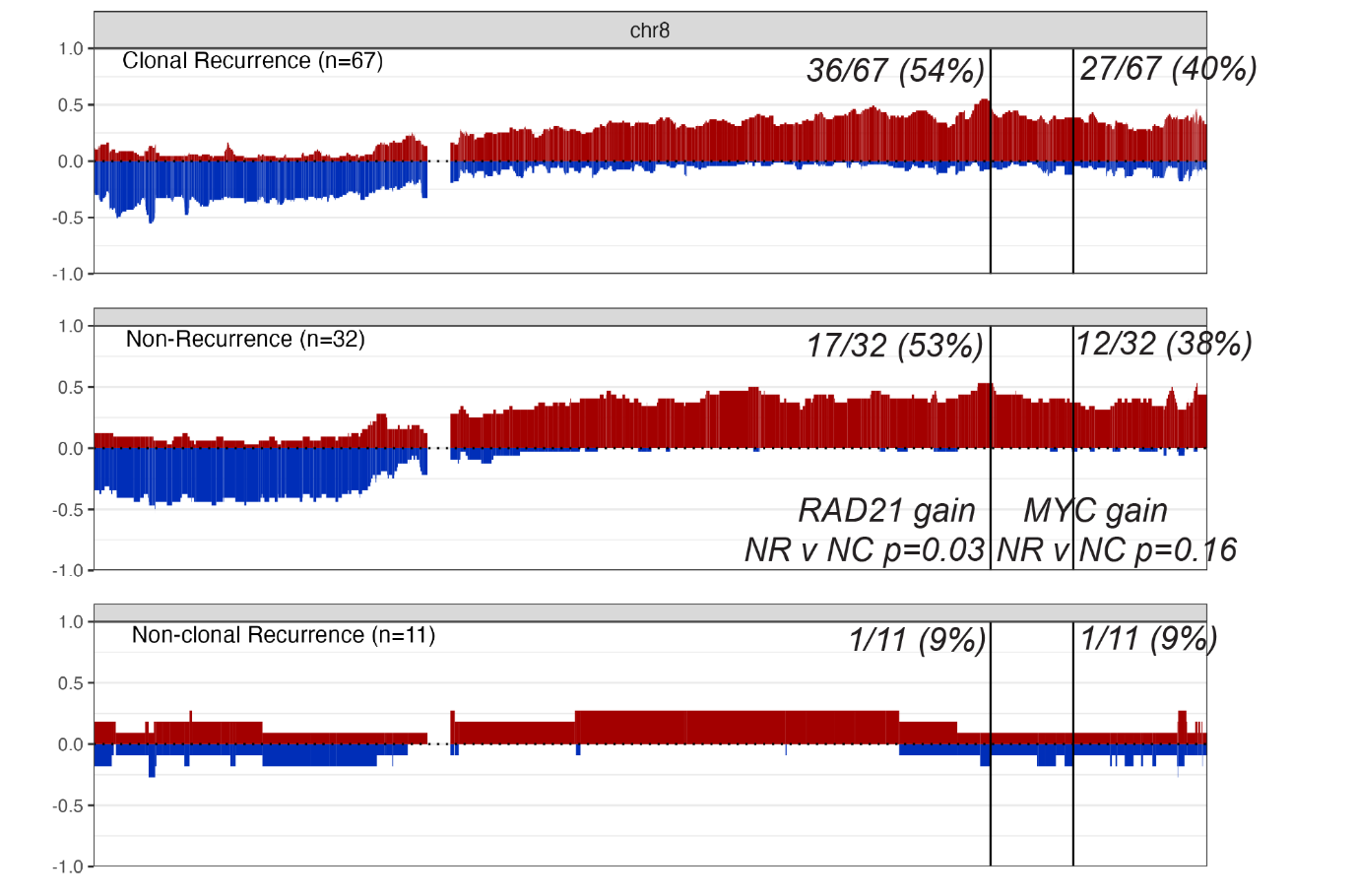


**Figure S15. CN frequency plot of chromosome 8** **highlighting *RAD21* (significantly different CN gain frequency between non-recurrent and non-clonal recurrence) and *MYC* (not different).** Red indicates gains and blue indicates losses. The *p* values are from two-tailed Fisher’s exact test. NR, non-recurrent; NC, non-clonal recurrence.

**
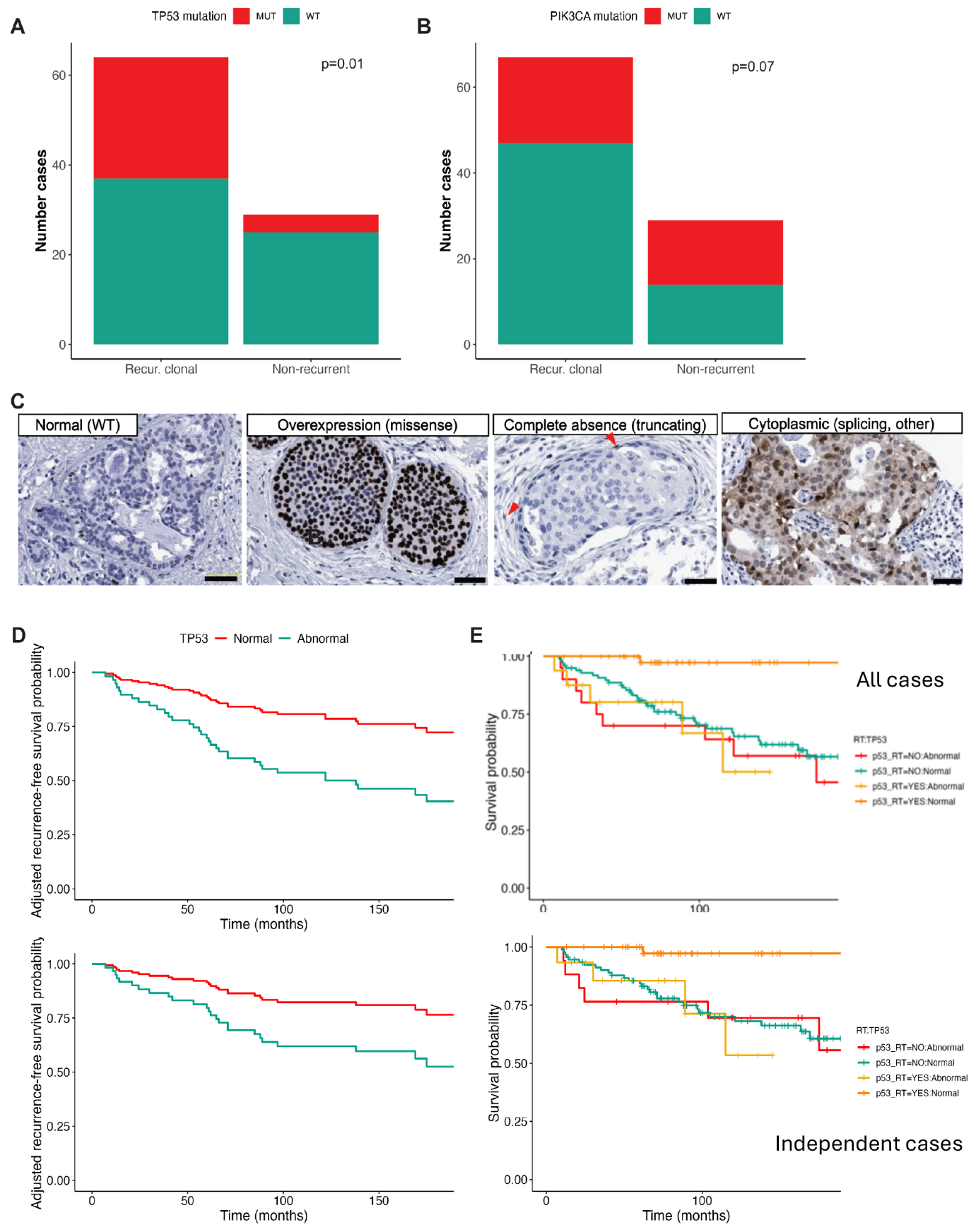
**

**Figure S16.** **Association of mutations with recurrence.** Number of cases with (A) *TP53* or (B) *PIK3CA* mutation. *p* value from Fisher’s exact test. WT, wild type; MUT, mutation. (C) TP53 immunohistochemistry staining patterns in DCIS: normal expression with heterogeneous staining intensities in <50% of cells; overexpression pattern; complete absence of TP53 with non-tumour cells showing normal staining as an internal control (arrowheads); and cytosolic staining. Black scale bar: 50 μm. (D) Ipsilateral recurrence-free survival curves of DCIS by TP53 status, adjusted for age, grade, ER, PR, HER2, and radiotherapy treatment. (E) Ipsilateral recurrence-free survival curves of DCIS, illustrating the interaction between TP53 (p53) and radiotherapy (RT). For panels D and E, the curves above are all cases, while those below exclude the ten cases that were part of the genetic cohort.

**
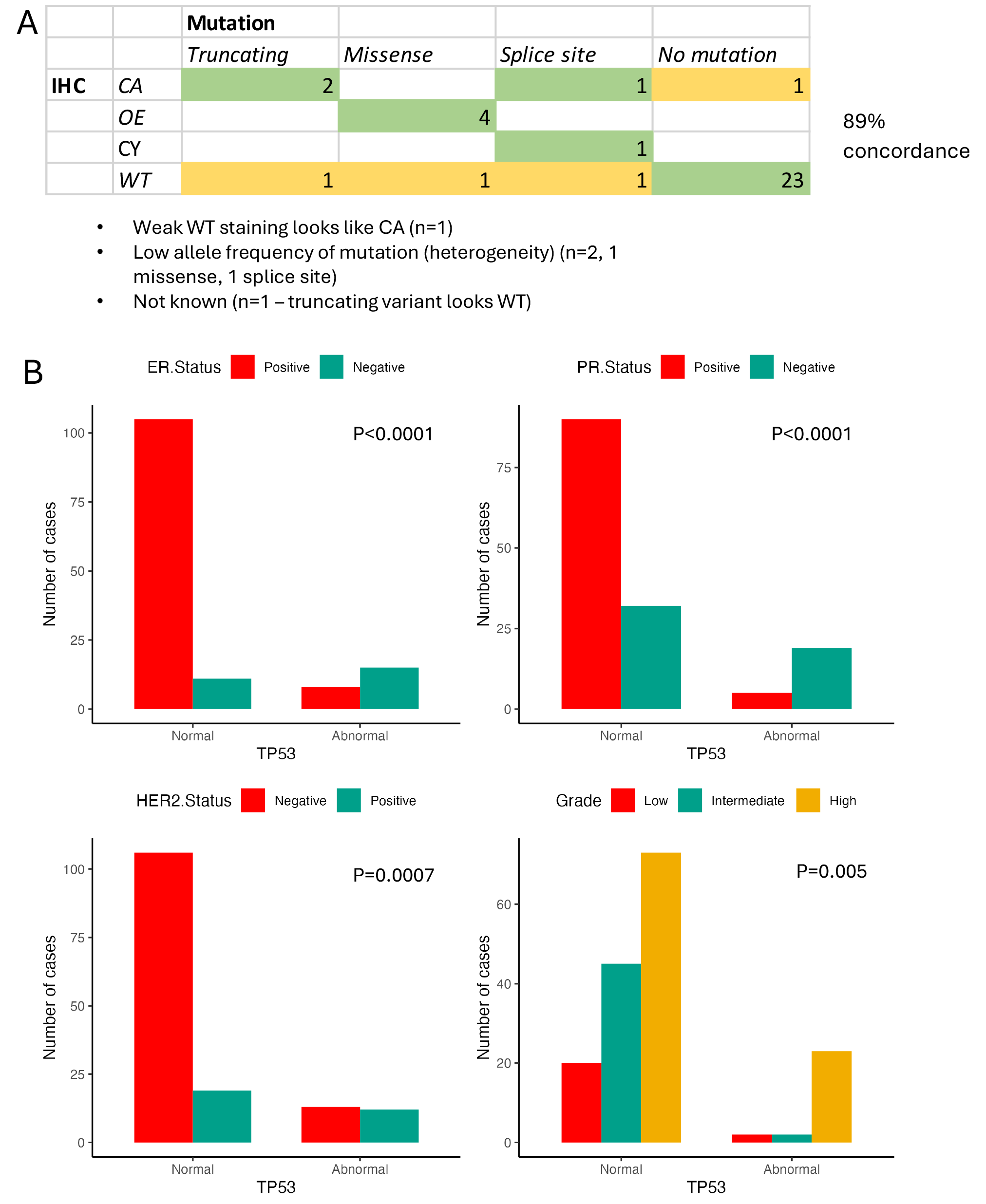
**

**Figure S17. TP53 immunohistochemistry.** (A) Concordance between immunohistochemistry (IHC) and mutation data. CA, complete absence; OE, overexpression; CY, cytosolic; WT, wild type (normal). Reasons for discordance are given below the table. (B) Association of TP53 staining result with histopathological features. *p* values from Fisher’s exact tests.


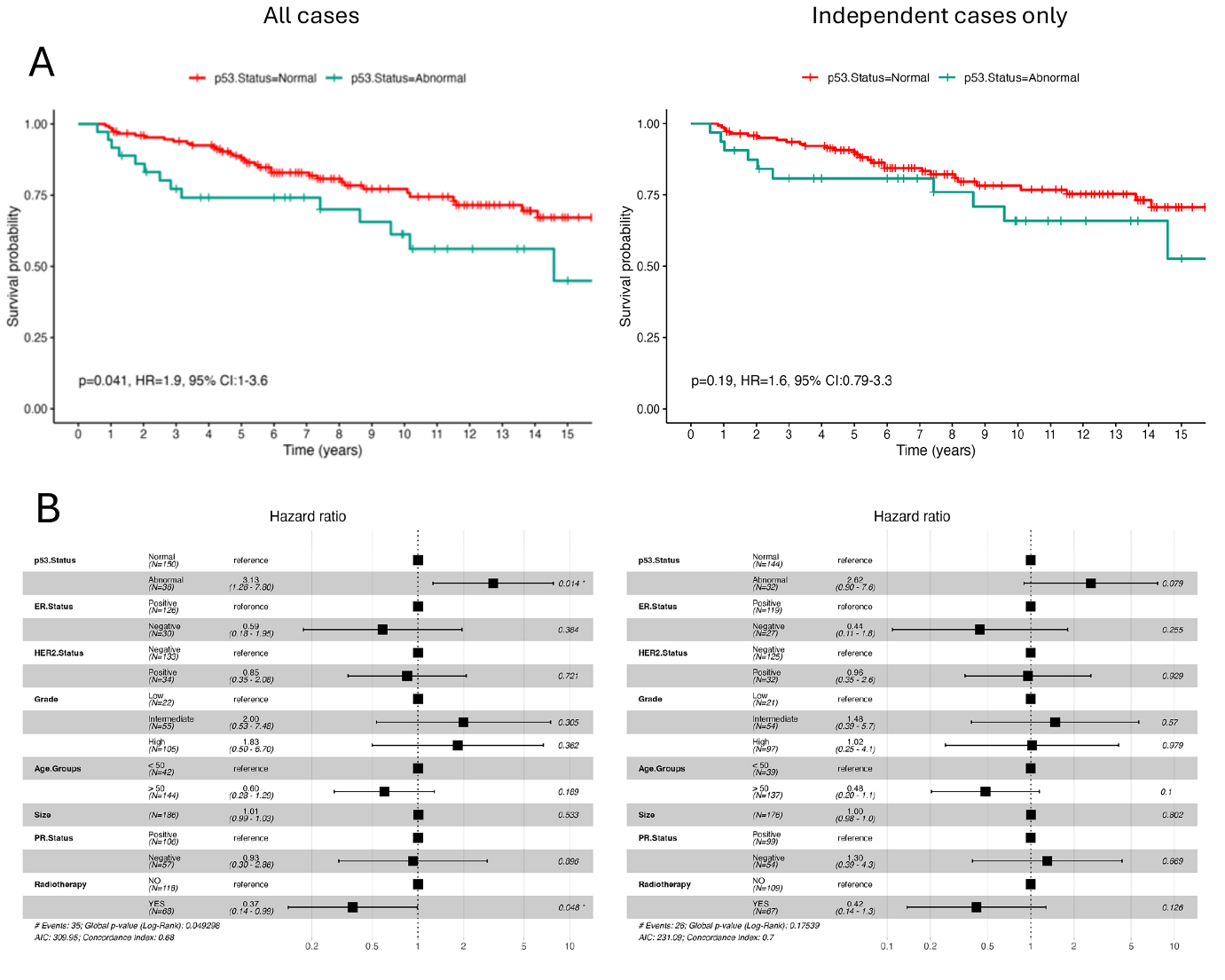


**Figure S18. TP53 staining association with recurrence with all cases (left) and with cases also present in the genetic cohort (*n* = 10) removed (right).** (A) Univariable Kaplan–Meier curve. (B) Multivariable Cox regression including the factors shown.

**Table S2.** List of genes on targeted sequencing panel

| **Gene** | **Group** | **Gene** | **Group** | **Gene** | **Group** |
| --- | --- | --- | --- | --- | --- |
| *AKT1* | Mutated | *AGMO* | DCIS CN | *B3GAT1* | TCGA & DCIS CN |
| *AKT2* | Mutated | *AURKA* | DCIS CN | *C1orf158* | TCGA & DCIS CN |
| *ATM* | Mutated | *BCAS4* | DCIS CN | *CCND1* | TCGA & DCIS CN |
| *BRCA2* | Mutated | *BCL11A* | DCIS CN | *CCNE1* | TCGA & DCIS CN |
| *CBFB* | Mutated | *BMP4* | DCIS CN | *MIR378C* | TCGA & DCIS CN |
| *CDH1* | Mutated | *MIR4251* | DCIS CN | ***IGF1R*** | TCGA & DCIS CN |
| *ESR1* | Mutated | ***CAMTA1*** | DCIS CN | *GABRB3* | TCGA & DCIS CN |
| *FGFR2* | Mutated | *MIR6078* | DCIS CN | *GREM1* | TCGA & DCIS CN |
| *NCOR1* | Mutated | *MIR5100* | DCIS CN | *MAP3K9* | TCGA & DCIS CN |
| *PIK3R1* | Mutated | *LINC00709* | DCIS CN | *RHOB* | TCGA & DCIS CN |
| *RUNX1* | Mutated | *MIR3162* | DCIS CN | *TNFAIP8L3* | TCGA & DCIS CN |
| *SPEN* | Mutated | ***NCOA3*** | DCIS CN | ***ZNF217*** | TCGA & DCIS CN |
| *TBX3* | Mutated | ***LARGE*** | DCIS CN | *BUB1B* | TCGA & DCIS CN |
| *TP53* | Mutated | *MIR4535* | DCIS CN | *EGFR* | TCGA & DCIS CN |
| *ARID1A* | Mutated | *CTNNA2* | DCIS CN | *AKT3* | TCGA CN |
| *MED12* | Mutated | *DDIT4* | DCIS CN | *CDKN2A* | TCGA CN |
| *NF1* | Mutated, DCIS CN | *DNA2* | DCIS CN | *ERBB2* | TCGA CN |
| *GATA3* | Mutated, TCGA, and DCIS CN | *EP300* | DCIS CN | *FGFR1* | TCGA CN |
| *BRCA1* | Mutated, TCGA CN | *GDF5* | DCIS CN | *HRAS* | TCGA CN |
| *FOXA1* | Mutated, TCGA CN | *GNAS* | DCIS CN | *NRAS* | TCGA CN |
| *KMT2C* | Mutated, TCGA CN | *HAUS5* | DCIS CN | *STK11* | TCGA CN |
| *MAP2K4* | Mutated, TCGA CN | *IDI1* | DCIS CN | *TBL1XR1* | TCGA CN |
| *MAP3K1* | Mutated, TCGA CN | *NEU3* | DCIS CN | *WT1* | TCGA CN |
| *PIK3CA* | Mutated, TCGA CN | *NF2* | DCIS CN | *ADAM7* | TCGA CN |
| *PTEN* | Mutated, TCGA CN | *NKAPL* | DCIS CN | *BAGE2* | TCGA CN |
| *RB1* | Mutated, TCGA CN | *FAS* | DCIS CN | *CDK10* | TCGA CN |
|  |  | *OPCML* | DCIS CN | ***ZMIZ1*** | TCGA CN |
|  |  | *OPRM1* | DCIS CN | *LINC01029* | TCGA CN |
|  |  | *PIP4K2A* | DCIS CN | *MIR587* | TCGA CN |
|  |  | *PTK6* | DCIS CN | ***CSMD1*** | TCGA CN |
|  |  | *SPHK1* | DCIS CN | ***PTPRD*** | TCGA CN |
|  |  | *VASH1* | DCIS CN | *DACT2* | TCGA CN |
|  |  | BLM | DCIS CN | *EPHA7* | TCGA CN |
|  |  |  |  | *FOXO3* | TCGA CN |
|  |  |  |  | *GAB2* | TCGA CN |
|  |  |  |  | *IKBKB* | TCGA CN |
|  |  |  |  | *ISOC2* | TCGA CN |
|  |  |  |  | *MCL1* | TCGA CN |
|  |  |  |  | *MDM2* | TCGA CN |
|  |  |  |  | *MYC* | TCGA CN |
|  |  |  |  | *PAK1* | TCGA CN |
|  |  |  |  | *BAP1* | TCGA CN |
|  |  |  |  | *LIG3* | TCGA CN |
|  |  |  |  | *PMS2* | TCGA CN |
|  |  |  |  | *PSPC1* | TCGA CN |
|  |  |  |  | *RPS6KB1* | TCGA CN |
|  |  |  |  | *TPD52* | TCGA CN |
|  |  |  |  | *TRIML1* | TCGA CN |

*Bold italics* indicates genes that only include partial coding sequence.

# **Table S3.** Differences by recurrence clonality status.

|  | **Non-recurrent** | **Clonal recurrence** | **Non-clonal recurrence** | ***p* value**  (test statistic)^†^ |
| --- | --- | --- | --- | --- |
|  | *n* = 32* | *n* = 67* | *n* = 11* |  |
| **Age, years** | 64.0 (43.0–73.0) | 60.0 (42.0–84.0) | 59.0 (31.0–78.0) | 0.8 (0.46) |
| Unknown | 0 | 2 | 1 |  |
| **Radiotherapy** | 11 / 17 (65%) | 12 / 62 (19%) | 1 / 10 (10%) | <0.001 |
| Unknown | 15 | 5 | 1 |  |
| **Grade** |  |  |  | 0.12 |
| High | 14 / 32 (44%) | 45 / 67 (67%) | 6 / 11 (55%) |  |
| Intermediate | 14 / 32 (44%) | 13 / 67 (19%) | 3 / 11 (27%) |  |
| Low | 4 / 32 (12.5%) | 9 / 67 (13%) | 2 / 11 (18%) |  |
| **Time to recurrence, years** | N/A | 3.3 (1.0–14.3) | 4.7 (1.3–9.1) |  |
| **Type of recurrence** |  |  |  | >0.9 |
| DCIS | 0 / 0 (NA%) | 30 / 67 (45%) | 5 / 11 (45%) |  |
| IBC | 0 / 0 (NA%) | 37 / 67 (55%) | 6 / 11 (55%) |  |
| Unknown | 32 | 0 | 0 |  |
| **Size, mm** | 15.0 (2.0–48.0) | 15.5 (1.8–80.0) | 7.6 (2.0–17.0) | 0.072 (5.28) |
| Unknown | 0 | 1 | 1 |  |

|  | **Non-recurrent** | **Clonal recurrence** | **Non-clonal recurrence** | ***p* value** (test |
| --- | --- | --- | --- | --- |
|  | *n* = 32* | *n* = 67* | *n* = 11* | statistic)^†^ |
| **ER status** |  |  |  | 0.4 |
| Negative | 4 / 32 (13%) | 16 / 66 (24%) | 1 / 9 (11%) |  |
| Positive | 28 / 32 (88%) | 50 / 66 (76%) | 8 / 9 (89%) |  |
| Unknown | 0 | 1 | 2 |  |
| **HER2 status** |  |  |  | 0.7 |
| Negative | 24 / 32 (75%) | 46 / 67 (69%) | 9 / 11 (82%) |  |
| Positive | 8 / 32 (25%) | 21 / 67 (31%) | 2 / 11 (18%) |  |
| ***TP53* mutation** |  |  |  | 0.019 |
| MUT | 4 / 29 (14%) | 27 / 64 (42%) | 3 / 10 (30%) |  |
| WT | 25 / 29 (86%) | 37 / 64 (58%) | 7 / 10 (70%) |  |
| Unknown | 0 | 3 | 1 |  |
| ***PIK3CA* mutation** |  |  |  | 0.16 |
| MUT | 15 / 29 (52%) | 20 / 64 (30%) | 3 / 10 (30%) |  |
| WT | 14 / 29 (48%) | 44 / 64 (70%) | 7 / 10 (70%) |  |
| Unknown | 0 | 3 | 1 |  |
| **FGA** | 0.2 (0.0–0.5) | 0.2 (0.0–0.6) | 0.0 (0.0–0.6) | 0.021 (7.8) |
| **Ploidy** | 2.0 (1.9–3.4) | 2.1 (1.9–4.1) | 2.1 (2.0–4.0) | 0.11 (4.4) |
| Unknown | 3 | 3 | 1 |  |
| **WGD** |  |  |  | 0.7 |
| No | 10 / 10 (100%) | 46 / 52 (88%) | 4 / 4 (100%) |  |
| Yes | 0 / 10 (0%) | 6 / 52 (12%) | 0 / 4 (0%) |  |
| Unknown | 22 | 15 | 7 |  |
| **Tumour lymphocytes** | 5.0 (1.0–30.0) | 10.0 (0.0–90.0) | 5.0 (3.0–5.0) | 0.3 (2.34) |
| Unknown | 5 | 18 | 8 |  |

*Median (minimum–maximum); *n* / *N* (%).

^†^Kruskal–Wallis rank-sum test; Fisher’s exact test.

FGA, fraction genome altered; WGD, whole genome duplication; MUT, mutated; WT, wild type.

**Table S5.** TP53 immunohistochemistry cohort by TP53 status.

| **Variable** | **Abnormal**, *n* = 36* | **Normal**, *n* = 150* | ***p* value**^†^ |
| --- | --- | --- | --- |
| **Age, years** |  |  | >0.9 |
| <50 | 8 / 36 (22%) | 34 / 150 (23%) |  |
| >50 | 28 / 36 (78%) | 116 / 150 (77%) |  |
| **Radiotherapy** | 16 / 36 (44%) | 52 / 150 (35%) | 0.3 |
| **Grade** |  |  | 0.018 |
| High | 27 / 34 (79%) | 78 / 148 (53%) |  |
| Intermediate | 5 / 34 (15%) | 50 / 148 (34%) |  |
| Low | 2 / 34 (5.9%) | 20 / 148 (14%) |  |
| Unknown | 2 | 2 |  |
| **Size** | 21.1 (13.4) | 19.6 (17.6) | 0.2 |
| Unknown | 3 | 8 |  |
| **ER** |  |  | <0.001 |
| Negative | 17 / 30 (57%) | 13 / 126 (10%) |  |
| Positive | 13 / 30 (43%) | 113 / 126 (90%) |  |
| Unknown | 6 | 24 |  |
| **PR** |  |  | <0.001 |
| Negative | 24 / 31 (77%) | 33 / 132 (25%) |  |
| Positive | 7 / 31 (23%) | 99 / 132 (75%) |  |
| Unknown | 5 | 18 |  |
| **HER2** |  |  | <0.001 |
| Negative | 18 / 32 (56%) | 115 / 135 (85%) |  |
| Positive | 14 / 32 (44%) | 20 / 135 (15%) |  |
| Unknown | 4 | 15 |  |
| **Recurrence** | 14 / 36 (39%) | 35 / 150 (23%) | 0.057 |
| **n* / *N* (%); mean (SD). | | | |
| ^†^Pearson’s *χ*^2^ test; Fisher’s exact test; Wilcoxon rank-sum test. | | | |

**Table S6.** TP53 immunohistochemistry cohort by recurrence status

| **Variable** | **Non-recurrent**  *n* = 137* | **Recurrent**  *n* = 49* | ***p* value**^†^ |  |
| --- | --- | --- | --- | --- |
| **Age, years** |  |  | <0.001 |  |
| <50 | 22 / 137 (16%) | 20 / 49 (41%) |  |  |
| >50 | 115 / 137 (84%) | 29 / 49 (59%) |  |  |
| **Radiotherapy** | 62 / 137 (45%) | 6 / 49 (12%) | <0.001 |  |
| **Grade** |  |  | 0.4 |  |
| High | 78 / 137 (57%) | 27 / 45 (60%) |  |  |
| Intermediate | 40 / 137 (29%) | 15 / 45 (33%) |  |  |
| Low | 19 / 137 (14%) | 3 / 45 (6.7%) |  |  |
| Unknown | 0 | 4 |  |  |
| **Size** | 19.7 (15.9) | 20.6 (19.8) | 0.7 |  |
| Unknown | 5 | 6 |  |  |
| **ER** |  |  | 0.8 |  |
| Negative | 21 / 112 (19%) | 9 / 44 (20%) |  |  |
| Positive | 91 / 112 (81%) | 35 / 44 (80%) |  |  |
| Unknown | 25 | 5 |  |  |
| **PR** |  |  | 0.3 |  |
| Negative | 39 / 119 (33%) | 18 / 44 (41%) |  |  |
| Positive | 80 / 119 (67%) | 26 / 44 (59%) |  |  |
| Unknown | 18 | 5 |  |  |
| **HER2** |  |  | 0.9 |  |
| Negative | 96 / 120 (80%) | 37 / 47 (79%) |  |  |
| Positive | 24 / 120 (20%) | 10 / 47 (21%) |  |  |
| Unknown | 17 | 2 |  |  |
| **p53.Status** |  |  | 0.057 |  |
| Abnormal | 22 / 137 (16%) | 14 / 49 (29%) |  |  |
| Normal | 115 / 137 (84%) | 35 / 49 (71%) |  |  |
| **n* / *N* (%); mean (SD). | | | | |
| ^†^Pearson’s *χ*^2^ test; Wilcoxon rank-sum test. | | | | |

**Table S7.** General linear model for *TP53* mutation

| **Variable** | **OR** | **95% CI** | **OR** | **95% CI** | **OR** | **95% CI** |
| --- | --- | --- | --- | --- | --- | --- |
| Intercept | 1.48 | 0.896–2.49 | 6.227 | 0.903–65.02 | 10.515 | 3.237–47.77 |
| *TP53* (mutation) | 4.56 | 1.55–16.84 | 6.995 | 1.114–74.39 | 5.252 | 1.068–42.41 |
| Grade (int.) | – | – | 0.226 | 0.038–1.079 | 0.260 | 0.051–1.157 |
| Grade (low) | – | – | 1.157 | 0.125–26.12 | 1.215 | 0.134–27.27 |
| Radiotherapy (yes) | – | – | 0.097 | 0.018–0.397 | 0.106 | 0.021–0.416 |
| ER (positive) | – | – | 1.952 | 0.201–17.67 | – | – |

OR, odds ratio.

**Table S8.** Imputed model for *TP53* mutation

| **Variable** | ***χ*^2^** | **d.f.** | ***p* value** | ***χ*^2^** | **d.f.** | ***p* value** |
| --- | --- | --- | --- | --- | --- | --- |
| *TP53* mutation | 3.06 | 1 | 0.08 | 3.76 | 1 | 0.05 |
| Grade | 4.53 | 2 | 0.10 | 4.5 | 2 | 0.10 |
| Radiotherapy | 7.07 | 1 | 0.008 | 7.07 | 1 | 0.008 |
| ER | 0.03 | 1 | 0.87 | – | – | – |
| **Total** | **13.64** | **5** | **0.018** | **13.7** | **4** | **0.008** |
| ***C*** | **0.77** |  |  | **0.769** |  |  |

d.f., degrees of freedom; *C*, concordance.

**Table S9.** Multivariable Cox regression results for p53 IHC

|  | **All cases** | **Removed overlapping** | **All cases** | **Removed overlapping** | **All cases** | **Removed overlapping** | **All cases** | **Removed overlapping** |
| --- | --- | --- | --- | --- | --- | --- | --- | --- |
| **Variable** | **OR (95% CI)** | **OR (95% CI)** | **OR (95% CI)** | **OR (95% CI)** | **OR (95% CI)** | **OR (95% CI)** | **OR (95% CI)** | **OR (95% CI)** |
| p53 abnormal | 3.13 (1.26–7.80) | 2.62 (0.90–7.64) | 1.82 (0.64–5.17) | 1.38 (0.39–4.96) | 1.59 (0.77–3.30) | 1.35 (0.57–3.20) | 0.88 (0.36–2.11) | 0.64 (0.20–1.95) |
| ER negative | 0.59 (0.18–1.95) | 0.44 (0.11–1.80) | 0.61 (0.18–2.04) | 0.44 (0.11–1.77) |  |  |  |  |
| HER2 positive | 0.85 (0.35–2.08) | 0.96 (0.35–2.62) | 0.94 (0.39–2.28) | 1.06 (0.39–2.87 |  |  |  |  |
| Size | 1.01 (0.99–1.03) | 1.00 (0.98–1.03) | 1.00 (0.98–1.03) | 1.00 (0.98–1.03) |  |  |  |  |
| PR negative | 0.93 (0.30–2.86) | 1.30 (0.39–4.31) | 0.88 (0.28–2.75) | 1.26 (0.38–4.22) |  |  |  |  |
| Grade int. | 1.996 (0.53–7.47) | 1.48 (0.39–5.65) | 1.61 (0.43–6.08) | 1.16 (0.30–4.50) | 2.29 (0.65–8.00) | 1.95 (0.55–6.88) | 2.05 (0.59–7.15) | 1.75 (0.50–6.15) |
| Grade high | 1.828 (0.50–6.70) | 1.02 (0.25–4.08) | 1.70 (0.46–6.28) | 0.93 (0.23–3.77) | 2.20 (0.65–7.44) | 1.60 (0.46–5.59) | 2.29 (0.67–7.76) | 1.68 (0.48–5.88) |
| Age >50 years | 0.60 (0.28–1.29) | 0.48 (0.20–1.15) | 0.497 (0.23–1.10) | 0.39 (0.16–0.96) | 0.44 (0.24–0.80) | 0.37 (0.19–0.73) | 0.40 (0.22–0.73) | 0.34 (0.17–0.66) |
| RT yes | 0.37 (0.14–0.99) | 0.42 (0.14–1.28) | 0.09 (0.01–0.71) | 0.12 (0.016–0.95) | 0.31 (0.13–0.75) | 0.33 (0.12–0.87) | 0.06 (0.008–0.45) | 0.07 (0.009–0.54) |
| p53 abnormal: RT yes |  |  | 16.45 (1.46–185.2) | 17.76 (1.31–240.6) |  |  | 25.87 (2.55–262.98) | 29.02 (2.50–338.2) |
| **LR *p* value** | **0.049** | **0.19** | **0.009** | **0.051** | **2.00E-04** | **0.001** | **5.00E-06** | **5.00E-05** |
| **Concordance** | **0.685** | **0.698** | **0.723** | **0.743** | **0.712** | **0.713** | **0.741** | **0.745** |
| *n* | 134 | 124 | 134 | 124 | 182 | 172 | 182 | 172 |
| *n* events | 35 | 26 | 35 | 26 | 45 | 36 | 45 | 36 |
